# Supplementary material for: Integrated transcriptome meta-analysis of pancreatic ductal adenocarcinoma and matched adjacent pancreatic tissues
Source: PeerJ. 2020 Oct 27;8:e10141. doi: 10.7717/peerj.10141 (PMC7597628; doi:10.7717/peerj.10141)

**Supp. Figure 1.** DGKH was found to be a prognostic marker for PDAC in three external GEO datasets (GSE21501, GSE50827, and GSE57495). Kaplan-Meier plots for DGKH created using PROGgeneV2. The patient cohorts were divided into two equal groups based on median expression for DGKH. P<0.05 was accepted as statistically significant.


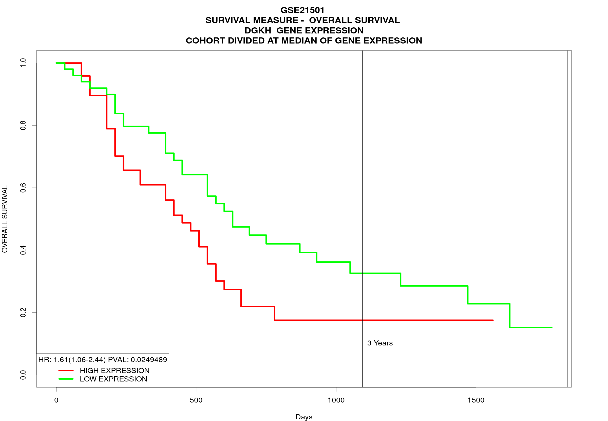

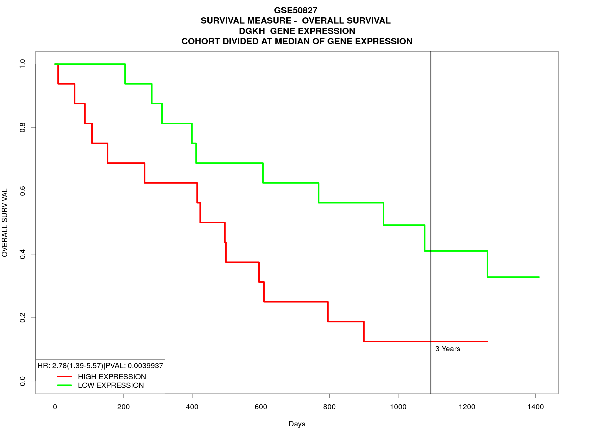

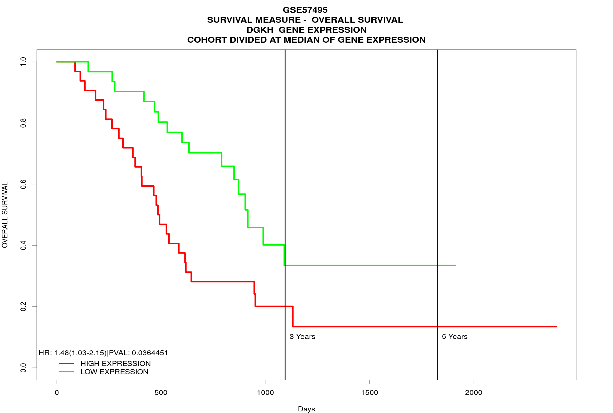

Supplement: Supplemental Information 1 — Kaplan-Meier plots for DGKH created using PROGgeneV2. The patient cohorts were divided into two equal groups based on median expression for DGKH. P<0.05 was accepted as statistically significant. [file peerj-08-10141-s001.docx]
