# Supplementary material for: Integrated transcriptome meta-analysis of pancreatic ductal adenocarcinoma and matched adjacent pancreatic tissues
Source: PeerJ. 2020 Oct 27;8:e10141. doi: 10.7717/peerj.10141 (PMC7597628; doi:10.7717/peerj.10141)
Supplement: Supplemental Information 2 — NS: p≥ 0.05, N/A; not available. [file peerj-08-10141-s002.docx]

**Supp. Table 1. The lists of up- and down-regulated genes in PDAC.**

***NS: p≥ 0.05,* N/A; not available.**

|  | *UP-REGULATED GENES*  *PDAC vs. Adjacent Tissues* |  | | | | *GENE EXPRESSION VALIDATION DATASETS*  *PDAC vs. Healthy*  *TCGA PAAD combined GTEx Data* |
| --- | --- | --- | --- | --- | --- | --- |
| *Gene symbol* | ***Gene name*** | ***Logratio combined*** | ***Fold change combined*** | ***p-value*** | ***FDR*** | ***p-value*** |
| POSTN | Periostin, osteoblast specific factor | 1,0004 | 10,009 | 0 | 0 | ≤0.001 |
| CEACAM5 | Carcinoembryonic antigen-related cell adhesion molecule 5 | 0,8855 | 7,683 | 0 | 0 | ≤0.001 |
| SLC6A14 | Solute carrier family 6 (amino acid transporter), member 14 | 0,8611 | 7,263 | 0 | 0 | ≤0.001 |
| CEACAM6 | Carcinoembryonic antigen-related cell adhesion molecule 6 (non-specific cross reacting antigen) | 0,8452 | 7,002 | 0 | 0 | ≤0.001 |
| SULF1 | Sulfatase 1 | 0,8347 | 6,835 | 0 | 0 | ≤0.001 |
| LAMC2 | Laminin subunit gamma 2 | 0,8279 | 6,728 | 0 | 0 | ≤0.001 |
| FN1 | Fibronectin 1 | 0,8083 | 6,432 | 0 | 0 | ≤0.001 |
| COL11A1 | Collagen, type XI, alpha 1 | 0,7918 | 6,191 | 0 | 0 | ≤0.001 |
| INHBA | Inhibin beta A | 0,7713 | 5,907 | 0 | 0 | ≤0.001 |
| VCAN | Versican | 0,7644 | 5,813 | 0 | 0 | ≤0.001 |
| CTSE | Cathepsin E | 0,7624 | 5,786 | 0 | 0 | ≤0.001 |
| COL10A1 | Collagen, type X, alpha 1 | 0,7496 | 5,618 | 0 | 0 | ≤0.001 |
| KRT19 | Keratin 19, type I | 0,7324 | 5,4 | 0 | 0 | ≤0.001 |
| ITGB6 | Integrin beta 6 | 0,6906 | 4,905 | 0 | 0 | ≤0.001 |
| THBS2 | Thrombospondin 2 | 0,6906 | 4,905 | 0 | 0 | ≤0.001 |
| COL8A1 | Collagen, type VIII, alpha 1 | 0,6815 | 4,802 | 0 | 0 | ≤0.001 |
| ITGA2 | Integrin, alpha 2 (CD49B, alpha 2 subunit of VLA-2 receptor) | 0,6798 | 4,784 | 0 | 0 | ≤0.001 |
| TSPAN1 | Tetraspanin 1 | 0,6766 | 4,749 | 0 | 0 | ≤0.001 |
| SERPINB5 | Serpin peptidase inhibitor, clade B (ovalbumin), member 5 | 0,661 | 4,582 | 0 | 0 | ≤0.001 |
| COL1A1 | Collagen, type I, alpha 1 | 0,6583 | 4,553 | 0 | 0 | ≤0.001 |
| TMPRSS4 | Transmembrane protease, serine 4 | 0,6547 | 4,516 | 0 | 0 | ≤0.001 |
| CST1 | Cystatin SN | 0,6546 | 4,515 | 0 | 0 | ≤0.001 |
| GABRP | Gamma-aminobutyric acid (GABA) A receptor, pi | 0,6497 | 4,464 | 0 | 0 | ≤0.001 |
| CDH11 | Cadherin 11, type 2, OB-cadherin (osteoblast) | 0,6478 | 4,444 | 0 | 0 | ≤0.001 |
| CLDN18 | Claudin 18 | 0,6433 | 4,398 | 0 | 0 | ≤0.001 |
| LAMB3 | Laminin subunit beta 3 | 0,6288 | 4,254 | 0 | 0 | ≤0.001 |
| COL5A2 | Collagen, type V, alpha 2 | 0,6278 | 4,244 | 0 | 0 | ≤0.001 |
| CTHRC1 | Collagen triple helix repeat containing 1 | 0,6273 | 4,24 | 0 | 0 | ≤0.001 |
| COL1A2 | Collagen, type I, alpha 2 | 0,6247 | 4,214 | 0 | 0 | ≤0.001 |
| FAP | Fibroblast activation protein alpha | 0,6232 | 4,199 | 0 | 0 | ≤0.001 |
| GALNT5 | Polypeptide N-acetylgalactosaminyltransferase 5 | 0,6154 | 4,125 | 0 | 0 | ≤0.001 |
| COL12A1 | Collagen, type XII, alpha 1 | 0,6014 | 3,994 | 0 | 0 | ≤0.001 |
| SLPI | Secretory leukocyte peptidase inhibitor | 0,5971 | 3,955 | 0 | 0 | ≤0.001 |
| MMP1 | Matrix metallopeptidase 1 | 0,5928 | 3,916 | 0 | 0 | ≤0.001 |
| S100P | S100 calcium binding protein P | 0,5925 | 3,913 | 0 | 0 | ≤0.001 |
| CXCL5 | Chemokine (C-X-C motif) ligand 5 | 0,5912 | 3,901 | 0 | 0 | ≤0.001 |
| TMC5 | Transmembrane channel like 5 | 0,5905 | 3,895 | 0 | 0 | ≤0.001 |
| CP | Ceruloplasmin (ferroxidase) | 0,5892 | 3,883 | 0 | 0 | ≤0.001 |
| KRT7 | Keratin 7, type II | 0,5866 | 3,861 | 0 | 0 | ≤0.001 |
| TCN1 | Transcobalamin 1 | 0,5837 | 3,834 | 0 | 0 | ≤0.001 |
| ANXA10 | Annexin A10 | 0,5784 | 3,788 | 0 | 0 | ≤0.001 |
| COL3A1 | Collagen, type III, alpha 1 | 0,5766 | 3,772 | 0 | 0 | ≤0.001 |
| DPCR1 | Diffuse panbronchiolitis critical region 1 | 0,5766 | 3,772 | 0 | 0 | ≤0.001 |
| ANTXR1 | Anthrax toxin receptor 1 | 0,5734 | 3,744 | 0 | 0 | ≤0.001 |
| COMP | Cartilage oligomeric matrix protein | 0,5708 | 3,722 | 0 | 0 | ≤0.001 |
| NOX4 | NADPH oxidase 4 | 0,5663 | 3,684 | 0 | 0 | ≤0.001 |
| MMP12 | Matrix metallopeptidase 12 | 0,5606 | 3,636 | 0 | 0 | ≤0.001 |
| AHNAK2 | AHNAK nucleoprotein 2 | 0,5595 | 3,627 | 0 | 0 | ≤0.001 |
| AGR2 | Anterior gradient 2, protein disulphide isomerase family member | 0,5575 | 3,61 | 0 | 0 | ≤0.001 |
| EDNRA | Endothelin receptor type A | 0,5502 | 3,55 | 0 | 0 | ≤0.001 |
| PLAC8 | Placenta specific 8 | 0,5495 | 3,544 | 0 | 0 | ≤0.001 |
| NTM | Neurotrimin | 0,5464 | 3,519 | 0 | 0 | ≤0.001 |
| OLR1 | Oxidized low density lipoprotein (lectin-like) receptor 1 | 0,5432 | 3,493 | 0 | 0 | ≤0.001 |
| IGFBP5 | Insulin like growth factor binding protein 5 | 0,5427 | 3,489 | 0 | 0 | ≤0.001 |
| - | RNA, 7SL, cytoplasmic 815, pseudogene | 0,5367 | 3,441 | 0 | 0 | - |
| EDIL3 | EGF-like repeats and discoidin I-like domains 3 | 0,5362 | 3,437 | 0 | 0 | ≤0.001 |
| TFF1 | Trefoil factor 1 | 0,5344 | 3,423 | 2,22E-16 | 1,27E-14 | ≤0.001 |
| LCN2 | Lipocalin 2 | 0,5278 | 3,371 | 0 | 0 | ≤0.001 |
| LEF1 | Lymphoid enhancer-binding factor 1 | 0,5232 | 3,336 | 0 | 0 | ≤0.001 |
| GREM1 | Gremlin 1, DAN family BMP antagonist | 0,523 | 3,334 | 0 | 0 | ≤0.001 |
| ANO1 | Anoctamin 1, calcium activated chloride channel | 0,519 | 3,303 | 0 | 0 | ≤0.001 |
| VSIG1 | V-set and immunoglobulin domain containing 1 | 0,5182 | 3,298 | 0 | 0 | ≤0.001 |
| CEMIP | Cell migration inducing protein, hyaluronan binding | 0,5138 | 3,264 | 0 | 0 | ≤0.001 |
| GPRC5A | G protein-coupled receptor, class C, group 5, member A | 0,5135 | 3,262 | 0 | 0 | ≤0.001 |
| NQO1 | NAD(P)H dehydrogenase, quinone 1 | 0,5107 | 3,241 | 0 | 0 | ≤0.001 |
| FNDC1 | Fibronectin type III domain containing 1 | 0,5076 | 3,218 | 0 | 0 | ≤0.001 |
| ANLN | Anillin actin binding protein | 0,506 | 3,206 | 0 | 0 | ≤0.001 |
| MMP7 | Matrix metallopeptidase 7 | 0,5047 | 3,197 | 0 | 0 | ≤0.001 |
| SLC6A6 | Solute carrier family 6 (neurotransmitter transporter), member 6 | 0,5046 | 3,196 | 0 | 0 | ≤0.001 |
| SFRP4 | Secreted frizzled-related protein 4 | 0,5024 | 3,18 | 2,00E-15 | 1,10E-13 | ≤0.001 |
| SEMA3C | Sema domain, immunoglobulin domain (Ig), short basic domain, secreted, (semaphorin) 3C | 0,5023 | 3,179 | 0 | 0 | ≤0.001 |
| IGFBP3 | Insulin like growth factor binding protein 3 | 0,5022 | 3,178 | 0 | 0 | ≤0.001 |
| SPARC | Secreted protein, acidic, cysteine-rich (osteonectin) | 0,4989 | 3,154 | 0 | 0 | ≤0.001 |
| KRT17 | Keratin 17, type I | 0,4968 | 3,139 | 0 | 0 | ≤0.001 |
| COL6A3 | Collagen, type VI, alpha 3 | 0,4939 | 3,118 | 0 | 0 | ≤0.001 |
| COL5A1 | Collagen, type V, alpha 1 | 0,4936 | 3,116 | 0 | 0 | ≤0.001 |
| SDR16C5 | Short chain dehydrogenase/reductase family 16C, member 5 | 0,493 | 3,111 | 0 | 0 | ≤0.001 |
| KYNU | Kynureninase | 0,4914 | 3,1 | 0 | 0 | ≤0.001 |
| PLAT | Plasminogen activator, tissue | 0,4898 | 3,089 | 0 | 0 | ≤0.001 |
| ADAM12 | ADAM metallopeptidase domain 12 | 0,4888 | 3,082 | 0 | 0 | ≤0.001 |
| GJB2 | Gap junction protein beta 2 | 0,4876 | 3,073 | 0 | 0 | ≤0.001 |
| TIMP1 | TIMP metallopeptidase inhibitor 1 | 0,4842 | 3,05 | 0 | 0 | ≤0.001 |
| TGM2 | Transglutaminase 2 | 0,4839 | 3,047 | 0 | 0 | ≤0.001 |
| MBOAT2 | Membrane bound O-acyltransferase domain containing 2 | 0,4837 | 3,046 | 0 | 0 | ≤0.001 |
| MYOF | Myoferlin | 0,4824 | 3,037 | 0 | 0 | ≤0.001 |
| PLAU | Plasminogen activator, urokinase | 0,4823 | 3,036 | 0 | 0 | ≤0.001 |
| LAMA3 | Laminin subunit alpha 3 | 0,4757 | 2,99 | 0 | 0 | ≤0.001 |
| ITGBL1 | Integrin beta like 1 | 0,4753 | 2,987 | 0 | 0 | ≤0.001 |
| MMP11 | Matrix metallopeptidase 11 | 0,4753 | 2,987 | 0 | 0 | ≤0.001 |
| FBN1 | Fibrillin 1 | 0,4735 | 2,975 | 0 | 0 | ≤0.001 |
| CD109 | CD109 molecule | 0,4711 | 2,959 | 0 | 0 | ≤0.001 |
| CCL20 | Chemokine (C-C motif) ligand 20 | 0,467 | 2,931 | 0 | 0 | ≤0.001 |
| NPR3 | Natriuretic peptide receptor 3 | 0,4644 | 2,914 | 0 | 0 | ≤0.001 |
| BGN | Biglycan | 0,4631 | 2,905 | 0 | 0 | ≤0.001 |
| SCEL | Sciellin | 0,461 | 2,891 | 0 | 0 | ≤0.001 |
| LOX | Lysyl oxidase | 0,4603 | 2,886 | 0 | 0 | ≤0.001 |
| DKK1 | Dickkopf WNT signaling pathway inhibitor 1 | 0,4593 | 2,88 | 0 | 0 | ≤0.001 |
| CAPG | Capping protein (actin filament), gelsolin-like | 0,4593 | 2,879 | 0 | 0 | ≤0.001 |
| IFI27 | Interferon, alpha-inducible protein 27 | 0,4574 | 2,867 | 0 | 0 | ≤0.001 |
| MATN3 | Matrilin 3 | 0,456 | 2,858 | 0 | 0 | ≤0.001 |
| SFRP2 | Secreted frizzled-related protein 2 | 0,4561 | 2,858 | 0 | 0 | ≤0.001 |
| FBXO32 | F-box protein 32 | 0,4559 | 2,857 | 0 | 0 | ≤0.001 |
| ECT2 | Epithelial cell transforming 2 | 0,4554 | 2,854 | 0 | 0 | ≤0.001 |
| RUNX2 | Runt-related transcription factor 2 | 0,4552 | 2,852 | 0 | 0 | ≤0.001 |
| CDH3 | Cadherin 3, type 1, P-cadherin (placental) | 0,4533 | 2,84 | 0 | 0 | ≤0.001 |
| WISP1 | WNT1 inducible signaling pathway protein 1 | 0,4529 | 2,837 | 0 | 0 | ≤0.001 |
| GCNT3 | Glucosaminyl (N-acetyl) transferase 3, mucin type | 0,4519 | 2,831 | 0 | 0 | ≤0.001 |
| IFI44L | Interferon induced protein 44 like | 0,4511 | 2,825 | 0 | 0 | ≤0.001 |
| TRIM29 | Tripartite motif containing 29 | 0,4511 | 2,825 | 0 | 0 | ≤0.001 |
| OLFML2B | Olfactomedin like 2B | 0,4505 | 2,822 | 0 | 0 | ≤0.001 |
| SULF2 | Sulfatase 2 | 0,4483 | 2,808 | 0 | 0 | ≤0.001 |
| GPX2 | Glutathione peroxidase 2 | 0,4479 | 2,805 | 0 | 0 | ≤0.001 |
| SRPX2 | Sushi-repeat containing protein, X-linked 2 | 0,4479 | 2,805 | 0 | 0 | ≤0.001 |
| SLC2A1 | Solute carrier family 2 (facilitated glucose transporter), member 1 | 0,4437 | 2,778 | 0 | 0 | ≤0.001 |
| FCGR3A | Fc fragment of igg, low affinity iiia, receptor (CD16a) | 0,4429 | 2,773 | 0 | 0 | ≤0.001 |
| RAB31 | RAB31, member RAS oncogene family | 0,4416 | 2,764 | 0 | 0 | ≤0.001 |
| TNFAIP6 | TNF alpha induced protein 6 | 0,4416 | 2,764 | 0 | 0 | ≤0.001 |
| AEBP1 | AE binding protein 1 | 0,4411 | 2,761 | 0 | 0 | ≤0.001 |
| RARRES1 | Retinoic acid receptor responder (tazarotene induced) 1 | 0,441 | 2,76 | 0 | 0 | ≤0.001 |
| ANXA8 | Annexin A8 | 0,44 | 2,754 | 0 | 0 | ≤0.001 |
| DUOX2 | Dual oxidase 2 | 0,439 | 2,748 | 5,22E-14 | 2,76E-12 | ≤0.001 |
| CTSK | Cathepsin K | 0,4384 | 2,744 | 0 | 0 | ≤0.001 |
| FERMT1 | Fermitin family member 1 | 0,4378 | 2,74 | 0 | 0 | ≤0.001 |
| ADAMTS12 | ADAM metallopeptidase with thrombospondin type 1 motif 12 | 0,4341 | 2,717 | 0 | 0 | ≤0.001 |
| TOP2A | Topoisomerase (DNA) II alpha | 0,4334 | 2,713 | 0 | 0 | ≤0.001 |
| SERPINB3 | Serpin peptidase inhibitor, clade B (ovalbumin), member 3 | 0,4309 | 2,697 | 0 | 0 | ≤0.001 |
| PCDH7 | Protocadherin 7 | 0,428 | 2,679 | 0 | 0 | ≤0.001 |
| ADGRF1 | Adhesion G protein-coupled receptor F1 | 0,4247 | 2,659 | 0 | 0 | ≤0.001 |
| COL17A1 | Collagen, type XVII, alpha 1 | 0,4246 | 2,658 | 0 | 0 | ≤0.001 |
| LAMP5 | Lysosomal associated membrane protein family member 5 | 0,4234 | 2,651 | 0 | 0 | ≤0.001 |
| SPP1 | Secreted phosphoprotein 1 | 0,4222 | 2,644 | 5,03E-13 | 2,60E-11 | ≤0.001 |
| ACSL5 | Acyl-coa synthetase long-chain family member 5 | 0,4196 | 2,628 | 0 | 0 | ≤0.001 |
| SFN | Stratifin | 0,4193 | 2,626 | 0 | 0 | ≤0.001 |
| ADAM28 | ADAM metallopeptidase domain 28 | 0,4184 | 2,621 | 0 | 0 | ≤0.001 |
| EGLN3 | Egl-9 family hypoxia-inducible factor 3 | 0,4159 | 2,606 | 0 | 0 | ≤0.001 |
| FXYD3 | FXYD domain containing ion transport regulator 3 | 0,4141 | 2,595 | 0 | 0 | ≤0.001 |
| CCL18 | Chemokine (C-C motif) ligand 18 | 0,4134 | 2,591 | 5,33E-15 | 2,90E-13 | ≤0.001 |
| CXCL8 | Chemokine (C-X-C motif) ligand 8 | 0,4132 | 2,589 | 2,22E-15 | 1,22E-13 | ≤0.001 |
| APOL1 | Apolipoprotein L1 | 0,4129 | 2,587 | 0 | 0 | ≤0.001 |
| NT5E | 5'-nucleotidase, ecto (CD73) | 0,4118 | 2,581 | 0 | 0 | ≤0.001 |
| DHRS9 | Dehydrogenase/reductase (SDR family) member 9 | 0,4109 | 2,576 | 0 | 0 | ≤0.001 |
| DPYSL3 | Dihydropyrimidinase like 3 | 0,41 | 2,571 | 0 | 0 | ≤0.001 |
| ACTA2 | Actin, alpha 2, smooth muscle, aorta | 0,4085 | 2,562 | 0 | 0 | ≤0.001 |
| ASPN | Asporin | 0,4063 | 2,548 | 0 | 0 | ≤0.001 |
| KRT6B | Keratin 6B, type II | 0,4057 | 2,545 | 0 | 0 | ≤0.001 |
| HEPH | Hephaestin | 0,4053 | 2,543 | 0 | 0 | ≤0.001 |
| LY75-CD302 | LY75-CD302 readthrough | 0,4046 | 2,539 | 0 | 0 | NS |
| MFAP5 | Microfibrillar associated protein 5 | 0,4045 | 2,538 | 1,55E-15 | 8,59E-14 | ≤0.001 |
| NRP2 | Neuropilin 2 | 0,4041 | 2,535 | 0 | 0 | ≤0.001 |
| ITGA3 | Integrin alpha 3 | 0,4022 | 2,525 | 0 | 0 | ≤0.001 |
| ETV1 | Ets variant 1 | 0,401 | 2,518 | 0 | 0 | ≤0.001 |
| CYP1B1 | Cytochrome P450, family 1, subfamily B, polypeptide 1 | 0,4006 | 2,516 | 3,13E-12 | 1,59E-10 | ≤0.001 |
| PLPP4 | Phospholipid phosphatase 4 | 0,3982 | 2,501 | 0 | 0 | ≤0.001 |
| GPNMB | Glycoprotein (transmembrane) nmb | 0,3968 | 2,493 | 0 | 0 | ≤0.001 |
| OSBPL3 | Oxysterol binding protein like 3 | 0,3967 | 2,493 | 0 | 0 | ≤0.001 |
| MET | MET proto-oncogene, receptor tyrosine kinase | 0,3965 | 2,492 | 0 | 0 | ≤0.001 |
| LUM | Lumican | 0,3955 | 2,486 | 0 | 0 | ≤0.001 |
| HK2 | Hexokinase 2 | 0,395 | 2,483 | 0 | 0 | ≤0.001 |
| ARNTL2 | Aryl hydrocarbon receptor nuclear translocator like 2 | 0,3931 | 2,472 | 0 | 0 | ≤0.001 |
| IGF2BP3 | Insulin like growth factor 2 mrna binding protein 3 | 0,3927 | 2,47 | 0 | 0 | ≤0.001 |
| KRT6A | Keratin 6A, type II | 0,3927 | 2,47 | 0 | 0 | ≤0.001 |
| MXRA5 | Matrix-remodelling associated 5 | 0,392 | 2,466 | 0 | 0 | ≤0.001 |
| LYZ | Lysozyme | 0,3914 | 2,463 | 1,08E-11 | 5,41E-10 | ≤0.001 |
| CXCR4 | Chemokine (C-X-C motif) receptor 4 | 0,3905 | 2,457 | 1,28E-12 | 6,54E-11 | ≤0.001 |
| CEACAM1 | Carcinoembryonic antigen-related cell adhesion molecule 1 (biliary glycoprotein) | 0,39 | 2,454 | 0 | 0 | ≤0.001 |
| SLC44A4 | Solute carrier family 44 member 4 | 0,3895 | 2,452 | 0 | 0 | ≤0.001 |
| TRIM31 | Tripartite motif containing 31 | 0,3887 | 2,447 | 6,73E-14 | 3,54E-12 | ≤0.001 |
| S100A6 | S100 calcium binding protein A6 | 0,3878 | 2,442 | 0 | 0 | ≤0.001 |
| PALLD | Palladin, cytoskeletal associated protein | 0,3876 | 2,441 | 0 | 0 | ≤0.001 |
| AREG | Amphiregulin | 0,3868 | 2,437 | 1,26E-13 | 6,58E-12 | ≤0.001 |
| GPX8 | Glutathione peroxidase 8 (putative) | 0,3861 | 2,433 | 0 | 0 | ≤0.001 |
| MMP2 | Matrix metallopeptidase 2 | 0,3855 | 2,43 | 0 | 0 | ≤0.001 |
| EFNB2 | Ephrin-B2 | 0,3852 | 2,428 | 0 | 0 | ≤0.001 |
| ARL4C | ADP ribosylation factor like gtpase 4C | 0,3832 | 2,417 | 0 | 0 | ≤0.001 |
| INPP4B | Inositol polyphosphate-4-phosphatase type II B | 0,3798 | 2,398 | 0 | 0 | ≤0.001 |
| LIPH | Lipase, member H | 0,3793 | 2,395 | 0 | 0 | ≤0.001 |
| KIF26B | Kinesin family member 26B | 0,3791 | 2,394 | 0 | 0 | ≤0.001 |
| MLPH | Melanophilin | 0,3791 | 2,394 | 0 | 0 | ≤0.001 |
| IFI44 | Interferon induced protein 44 | 0,3775 | 2,385 | 0 | 0 | ≤0.001 |
| PLXDC2 | Plexin domain containing 2 | 0,3775 | 2,385 | 0 | 0 | ≤0.001 |
| UGT1A1 | UDP glucuronosyltransferase 1 family, polypeptide A1 | 0,3762 | 2,378 | 2,71E-14 | 1,44E-12 | NS |
| ADAM9 | ADAM metallopeptidase domain 9 | 0,376 | 2,377 | 0 | 0 | ≤0.001 |
| CRP | C-reactive protein, pentraxin-related | 0,376 | 2,377 | 2,19E-10 | 1,07E-08 | ≤0.001 |
| IL1RN | Interleukin 1 receptor antagonist | 0,3759 | 2,376 | 0 | 0 | ≤0.001 |
| MOXD1 | Monooxygenase, DBH-like 1 | 0,3758 | 2,376 | 0 | 0 | ≤0.001 |
| SYTL2 | Synaptotagmin like 2 | 0,3749 | 2,371 | 0 | 0 | ≤0.001 |
| EFNA5 | Ephrin-A5 | 0,3742 | 2,367 | 0 | 0 | ≤0.001 |
| ASPM | Abnormal spindle microtubule assembly | 0,3732 | 2,362 | 0 | 0 | ≤0.001 |
| TGFBI | Transforming growth factor beta induced | 0,3723 | 2,357 | 0 | 0 | ≤0.001 |
| CALD1 | Caldesmon 1 | 0,3717 | 2,353 | 0 | 0 | ≤0.001 |
| PXDN | Peroxidasin | 0,3703 | 2,346 | 0 | 0 | ≤0.001 |
| NMU | Neuromedin U | 0,3702 | 2,345 | 0 | 0 | ≤0.001 |
| EPSTI1 | Epithelial stromal interaction 1 (breast) | 0,3698 | 2,343 | 0 | 0 | ≤0.001 |
| TMEM45B | Transmembrane protein 45B | 0,3694 | 2,341 | 1,53E-13 | 8,00E-12 | ≤0.001 |
| JUP | Junction plakoglobin | 0,3681 | 2,334 | 0 | 0 | ≤0.001 |
| LTBP1 | Latent transforming growth factor beta binding protein 1 | 0,3674 | 2,33 | 0 | 0 | ≤0.001 |
| CORIN | Corin, serine peptidase | 0,3663 | 2,324 | 0 | 0 | ≤0.001 |
| OSBPL10 | Oxysterol binding protein like 10 | 0,366 | 2,323 | 0 | 0 | ≤0.001 |
| ANXA2 | Annexin A2 | 0,3655 | 2,32 | 0 | 0 | ≤0.001 |
| CENPF | Centromere protein F | 0,3654 | 2,32 | 0 | 0 | ≤0.001 |
| LRRN1 | Leucine rich repeat neuronal 1 | 0,3654 | 2,319 | 0 | 0 | ≤0.001 |
| RGS1 | Regulator of G-protein signaling 1 | 0,3649 | 2,317 | 7,99E-15 | 4,32E-13 | ≤0.001 |
| XAF1 | XIAP associated factor 1 | 0,3646 | 2,316 | 0 | 0 | ≤0.001 |
| SLC16A4 | Solute carrier family 16 member 4 | 0,3646 | 2,315 | 0 | 0 | ≤0.001 |
| ANTXR2 | Anthrax toxin receptor 2 | 0,364 | 2,312 | 0 | 0 | ≤0.001 |
| EPHA4 | EPH receptor A4 | 0,3634 | 2,309 | 0 | 0 | ≤0.001 |
| TPM2 | Tropomyosin 2 (beta) | 0,3634 | 2,309 | 0 | 0 | ≤0.001 |
| GBP1 | Guanylate binding protein 1, interferon-inducible | 0,3628 | 2,306 | 6,66E-16 | 3,75E-14 | ≤0.001 |
| MTMR11 | Myotubularin related protein 11 | 0,3622 | 2,302 | 0 | 0 | ≤0.001 |
| RSAD2 | Radical S-adenosyl methionine domain containing 2 | 0,3614 | 2,298 | 0 | 0 | ≤0.001 |
| MUC13 | Mucin 13, cell surface associated | 0,3597 | 2,29 | 2,07E-08 | 9,75E-07 | ≤0.001 |
| NNMT | Nicotinamide N-methyltransferase | 0,3599 | 2,29 | 4,44E-16 | 2,53E-14 | ≤0.001 |
| IRAK3 | Interleukin 1 receptor associated kinase 3 | 0,3594 | 2,288 | 0 | 0 | ≤0.001 |
| DDX60 | DEAD (Asp-Glu-Ala-Asp) box polypeptide 60 | 0,3587 | 2,284 | 0 | 0 | ≤0.001 |
| MALL | Mal, T-cell differentiation protein-like | 0,3583 | 2,282 | 0 | 0 | ≤0.001 |
| SPOCK1 | Sparc/osteonectin, cwcv and kazal-like domains proteoglycan (testican) 1 | 0,3576 | 2,278 | 0 | 0 | ≤0.001 |
| DCBLD2 | Discoidin, CUB and LCCL domain containing 2 | 0,3566 | 2,273 | 0 | 0 | ≤0.001 |
| THY1 | Thy-1 cell surface antigen | 0,3566 | 2,273 | 0 | 0 | ≤0.001 |
| COL4A1 | Collagen, type IV, alpha 1 | 0,3551 | 2,265 | 0 | 0 | ≤0.001 |
| ITGB4 | Integrin beta 4 | 0,3539 | 2,259 | 0 | 0 | ≤0.001 |
| HMCN1 | Hemicentin 1 | 0,3527 | 2,253 | 0 | 0 | ≤0.001 |
| PRDM1 | PR domain containing 1, with ZNF domain | 0,3523 | 2,251 | 0 | 0 | ≤0.001 |
| HOPX | HOP homeobox | 0,3522 | 2,25 | 2,22E-16 | 1,27E-14 | ≤0.001 |
| CST2 | Cystatin SA | 0,3511 | 2,244 | 0 | 0 | ≤0.001 |
| PRRX1 | Paired related homeobox 1 | 0,3509 | 2,243 | 0 | 0 | ≤0.001 |
| LOXL2 | Lysyl oxidase like 2 | 0,3487 | 2,232 | 0 | 0 | ≤0.001 |
| MICAL2 | Microtubule associated monooxygenase, calponin and LIM domain containing 2 | 0,3488 | 2,232 | 0 | 0 | ≤0.001 |
| ANKRD22 | Ankyrin repeat domain 22 | 0,3486 | 2,231 | 1,27E-13 | 6,64E-12 | ≤0.001 |
| STYK1 | Serine/threonine/tyrosine kinase 1 | 0,3486 | 2,231 | 0 | 0 | ≤0.001 |
| FOXQ1 | Forkhead box Q1 | 0,3482 | 2,23 | 0 | 0 | ≤0.001 |
| ALOX5AP | Arachidonate 5-lipoxygenase-activating protein | 0,3468 | 2,223 | 0 | 0 | ≤0.001 |
| CTSB | Cathepsin B | 0,3469 | 2,223 | 0 | 0 | ≤0.001 |
| SLC22A3 | Solute carrier family 22 (organic cation transporter), member 3 | 0,346 | 2,218 | 0 | 0 | ≤0.001 |
| OAS2 | 2'-5'-oligoadenylate synthetase 2 | 0,3458 | 2,217 | 0 | 0 | ≤0.001 |
| LAMA4 | Laminin subunit alpha 4 | 0,3457 | 2,216 | 0 | 0 | ≤0.001 |
| IFI16 | Interferon, gamma-inducible protein 16 | 0,3447 | 2,212 | 2,22E-16 | 1,27E-14 | ≤0.001 |
| AK4 | Adenylate kinase 4 | 0,3446 | 2,211 | 0 | 0 | N/A  (Logsdon Pancreas,  1.16E-5) |
| ENO2 | Enolase 2 (gamma, neuronal) | 0,3439 | 2,207 | 0 | 0 | ≤0.001 |
| SPON1 | Spondin 1, extracellular matrix protein | 0,3434 | 2,205 | 3,83E-13 | 1,99E-11 | ≤0.001 |
| CD55 | CD55 molecule, decay accelerating factor for complement (Cromer blood group) | 0,3428 | 2,202 | 0 | 0 | ≤0.001 |
| - | RNA, 5S ribosomal pseudogene 385 | 0,3428 | 2,202 | 7,55E-15 | 4,09E-13 | - |
| SAMD9 | Sterile alpha motif domain containing 9 | 0,3428 | 2,202 | 0 | 0 | ≤0.001 |
| TSPAN8 | Tetraspanin 8 | 0,3427 | 2,201 | 1,37E-11 | 6,84E-10 | ≤0.001 |
| OLFM4 | Olfactomedin 4 | 0,3413 | 2,194 | 6,55E-06 | 0,000299 | ≤0.001 |
| TMSB10 | Thymosin beta 10 | 0,3412 | 2,194 | 0 | 0 | ≤0.001 |
| DGKH | Diacylglycerol kinase, eta | 0,3404 | 2,19 | 0 | 0 | ≤0.001 |
| SQLE | Squalene epoxidase | 0,3404 | 2,19 | 0 | 0 | ≤0.001 |
| MAP4K4 | Mitogen-activated protein kinase kinase kinase kinase 4 | 0,3398 | 2,187 | 0 | 0 | ≤0.001 |
| FGD6 | FYVE, rhogef and PH domain containing 6 | 0,3391 | 2,183 | 0 | 0 | ≤0.001 |
| KRT23 | Keratin 23, type I | 0,3389 | 2,182 | 0 | 0 | ≤0.001 |
| OAS1 | 2'-5'-oligoadenylate synthetase 1 | 0,3387 | 2,181 | 0 | 0 | ≤0.001 |
| MUC16 | Mucin 16, cell surface associated | 0,338 | 2,178 | 0 | 0 | ≤0.001 |
| DLGAP5 | Discs, large (Drosophila) homolog-associated protein 5 | 0,3368 | 2,172 | 0 | 0 | ≤0.001 |
| MUC4 | Mucin 4, cell surface associated | 0,3368 | 2,172 | 0 | 0 | ≤0.001 |
| FRMD6  ^(C14orf31)^ | FERM domain containing 6 | 0,3367 | 2,171 | 0 | 0 | ≤0.001 |
| SHISA2 | Shisa family member 2 | 0,3367 | 2,171 | 0 | 0 | ≤0.001 |
| HN1 | Jupiter microtubule associated homolog 1 | 0,3351 | 2,163 | 0 | 0 | ≤0.001 |
| S100A14 | S100 calcium binding protein A14 | 0,3331 | 2,153 | 0 | 0 | ≤0.001 |
| PFKP | Phosphofructokinase, platelet | 0,3326 | 2,151 | 0 | 0 | ≤0.001 |
| COL4A2 | Collagen, type IV, alpha 2 | 0,3319 | 2,148 | 0 | 0 | ≤0.001 |
| PLEK2 | Pleckstrin 2 | 0,3321 | 2,148 | 0 | 0 | ≤0.001 |
| LGALS1 | Lectin, galactoside-binding, soluble, 1 | 0,3317 | 2,146 | 0 | 0 | ≤0.001 |
| GABBR1 | Gamma-aminobutyric acid (GABA) B receptor, 1 | 0,3309 | 2,142 | 1,84E-12 | 9,36E-11 | NS |
| SERPINA1 | Serpin peptidase inhibitor, clade A (alpha-1 antiproteinase, antitrypsin), member 1 | 0,3302 | 2,139 | 0 | 0 | ≤0.001 |
| MIA | Melanoma inhibitory activity | 0,3298 | 2,137 | 0 | 0 | ≤0.001 |
| BCAS1 | Breast carcinoma amplified sequence 1 | 0,3297 | 2,136 | 0 | 0 | ≤0.001 |
| C15orf48 | Chromosome 15 open reading frame 48 | 0,3296 | 2,136 | 0 | 0 | ≤0.001 |
| SGPP2 | Sphingosine-1-phosphate phosphatase 2 | 0,3293 | 2,135 | 0 | 0 | ≤0.001 |
| MMP14 | Matrix metallopeptidase 14 (membrane-inserted) | 0,3292 | 2,134 | 0 | 0 | ≤0.001 |
| MSLN | Mesothelin | 0,3291 | 2,134 | 0 | 0 | ≤0.001 |
| MAST2 | Microtubule associated serine/threonine kinase 2 | 0,329 | 2,133 | 1,24E-08 | 5,90E-07 | ≤0.001 |
| PMEPA1 | Prostate transmembrane protein, androgen induced 1 | 0,3289 | 2,133 | 0 | 0 | ≤0.001 |
| F5 | Coagulation factor V (proaccelerin, labile factor) | 0,3288 | 2,132 | 0 | 0 | ≤0.001 |
| EPYC | Epiphycan | 0,3289 | 2,132 | 3,50E-11 | 1,72E-09 | ≤0.001 |
| ADAMTS2 | ADAM metallopeptidase with thrombospondin type 1 motif 2 | 0,3271 | 2,124 | 0 | 0 | ≤0.001 |
| PLA2R1 | Phospholipase A2 receptor 1 | 0,3269 | 2,123 | 0 | 0 | ≤0.001 |
| S100A16 | S100 calcium binding protein A16 | 0,3269 |  | 0 | 0 | ≤0.001 |
| RHBDL2 | Rhomboid, veinlet-like 2 (Drosophila) | 0,3255 | 2,116 | 0 | 0 | ≤0.001 |
| HOXB3 | Homeobox B3 | 0,3243 | 2,11 | 0 | 0 | ≤0.001 |
| FXYD5 | FXYD domain containing ion transport regulator 5 | 0,3234 | 2,106 | 0 | 0 | ≤0.001 |
| NCF2 | Neutrophil cytosolic factor 2 | 0,3232 | 2,105 | 0 | 0 | ≤0.001 |
| ARHGAP42 | Rho gtpase activating protein 42 | 0,3228 | 2,103 | 0 | 0 | ≤0.001 |
| MPZL2 | Myelin protein zero-like 2 | 0,3226 | 2,102 | 0 | 0 | ≤0.001 |
| PLAUR | Plasminogen activator, urokinase receptor | 0,3224 | 2,101 | 0 | 0 | ≤0.001 |
| PDGFRB | Platelet-derived growth factor receptor, beta polypeptide | 0,3225 | 2,101 | 0 | 0 | ≤0.001 |
| CCNB1 | Cyclin B1 | 0,3217 | 2,097 | 0 | 0 | ≤0.001 |
| PKM | Pyruvate kinase, muscle | 0,3216 | 2,097 | 0 | 0 | ≤0.001 |
| CXCL10 | Chemokine (C-X-C motif) ligand 10 | 0,3203 | 2,091 | 2,07E-14 | 1,10E-12 | ≤0.001 |
| IL1RAP | Interleukin 1 receptor accessory protein | 0,3204 | 2,091 | 0 | 0 | ≤0.001 |
| S100A4 | S100 calcium binding protein A4 | 0,3197 | 2,088 | 0 | 0 | ≤0.001 |
| SERPINH1 | Serpin peptidase inhibitor, clade H (heat shock protein 47), member 1, (collagen binding protein 1) | 0,3197 | 2,088 | 0 | 0 | ≤0.001 |
| TPX2 | TPX2, microtubule-associated | 0,3198 | 2,088 | 0 | 0 | ≤0.001 |
| CFH | Complement factor H | 0,3193 | 2,086 | 1,33E-15 | 7,42E-14 | ≤0.001 |
| IGFL2 | IGF like family member 2 | 0,3191 | 2,085 | 0 | 0 | ≤0.001 |
| FER1L6 | Fer-1-like family member 6 | 0,3188 | 2,084 | 1,15E-12 | 5,89E-11 | ≤0.001 |
| MUC17 | Mucin 17, cell surface associated | 0,3189 | 2,084 | 7,71E-08 | 3,62E-06 | ≤0.001 |
| NUAK1 | NUAK family, SNF1-like kinase, 1 | 0,3187 | 2,083 | 0 | 0 | ≤0.001 |
| ENTPD1 | Ectonucleoside triphosphate diphosphohydrolase 1 | 0,3186 | 2,082 | 0 | 0 | ≤0.001 |
| KCNK1 | Potassium channel, two pore domain subfamily K, member 1 | 0,3183 | 2,081 | 0 | 0 | ≤0.001 |
| LAPTM5 | Lysosomal protein transmembrane 5 | 0,3176 | 2,078 | 1,35E-14 | 7,31E-13 | ≤0.001 |
| PDPN | Podoplanin | 0,3177 | 2,078 | 0 | 0 | ≤0.001 |
| CENPK | Centromere protein K | 0,3172 | 2,076 | 0 | 0 | ≤0.001 |
| S100A11 | S100 calcium binding protein A11 | 0,317 | 2,075 | 0 | 0 | ≤0.001 |
| SKAP2 | Src kinase associated phosphoprotein 2 | 0,317 | 2,075 | 0 | 0 | ≤0.001 |
| ANXA1 | Annexin A1 | 0,3162 | 2,071 | 7,33E-13 | 3,77E-11 | ≤0.001 |
| PI3 | Peptidase inhibitor 3, skin-derived | 0,3162 | 2,071 | 0 | 0 | ≤0.001 |
| IL1R2 | Interleukin 1 receptor, type II | 0,316 | 2,07 | 6,66E-16 | 3,75E-14 | ≤0.001 |
| TNC | Tenascin C | 0,3154 | 2,067 | 2,86E-10 | 1,40E-08 | ≤0.001 |
| XDH | Xanthine dehydrogenase | 0,3153 | 2,067 | 2,09E-14 | 1,11E-12 | ≤0.001 |
| LPCAT2 | Lysophosphatidylcholine acyltransferase 2 | 0,3151 | 2,066 | 0 | 0 | ≤0.001 |
| MRC1 | Mannose receptor, C type 1 | 0,3145 | 2,063 | 2,21E-12 | 1,12E-10 | ≤0.001 |
| PLXNC1 | Plexin C1 | 0,314 | 2,061 | 0 | 0 | ≤0.001 |
| INSL5 | Insulin like 5 | 0,3138 | 2,06 | 7,13E-11 | 3,51E-09 | NS |
| MKI67 | Marker of proliferation Ki-67 | 0,3139 | 2,06 | 0 | 0 | ≤0.001 |
| SFTA2 | Surfactant associated 2 | 0,3138 | 2,06 | 0 | 0 | ≤0.001 |
| GLT8D2 | Glycosyltransferase 8 domain containing 2 | 0,3134 | 2,058 | 0 | 0 | ≤0.001 |
| LMO7 | LIM domain 7 | 0,3135 | 2,058 | 0 | 0 | ≤0.001 |
| PTPRR | Protein tyrosine phosphatase, receptor type, R | 0,3135 | 2,058 | 0 | 0 | ≤0.001 |
| MSR1 | Macrophage scavenger receptor 1 | 0,3124 | 2,053 | 8,88E-16 | 4,96E-14 | ≤0.001 |
| MELK | Maternal embryonic leucine zipper kinase | 0,3124 | 2,053 | 0 | 0 | ≤0.001 |
| AHR | Aryl hydrocarbon receptor | 0,3123 | 2,052 | 0 | 0 | ≤0.001 |
| BICD1 | Bicaudal D homolog 1 (Drosophila) | 0,3122 | 2,052 | 0 | 0 | ≤0.001 |
| KCNN4 | Potassium channel, calcium activated intermediate/small conductance subfamily N alpha, member 4 | 0,3122 | 2,052 | 0 | 0 | ≤0.001 |
| SGIP1 | SH3-domain GRB2-like (endophilin) interacting protein 1 | 0,3114 | 2,049 | 0 | 0 | ≤0.001 |
| PLA2G7 | Phospholipase A2 group VII | 0,3105 | 2,044 | 1,47E-14 | 7,85E-13 | ≤0.001 |
| PADI1 | Peptidyl arginine deiminase, type I | 0,3099 | 2,041 | 0 | 0 | ≤0.001 |
| SERPINB2 | Serpin peptidase inhibitor, clade B (ovalbumin), member 2 | 0,3098 | 2,041 | 8,64E-14 | 4,53E-12 | ≤0.001 |
| TRIM59 | Tripartite motif containing 59 | 0,3099 | 2,041 | 0 | 0 | ≤0.001 |
| BST2 | Bone marrow stromal cell antigen 2 | 0,3085 | 2,034 | 0 | 0 | ≤0.001 |
| GNB4 | Guanine nucleotide binding protein (G protein), beta polypeptide 4 | 0,3081 | 2,033 | 0 | 0 | ≤0.001 |
| CEP170 | Centrosomal protein 170kda | 0,3079 | 2,032 | 0 | 0 | ≤0.001 |
| FAM83D | Family with sequence similarity 83 member D | 0,3075 | 2,03 | 0 | 0 | ≤0.001 |
| DACT1 | Dishevelled-binding antagonist of beta-catenin 1 | 0,3072 | 2,029 | 0 | 0 | ≤0.001 |
| GPR87 | G protein-coupled receptor 87 | 0,3073 | 2,029 | 0 | 0 | ≤0.001 |
| MGLL | Monoglyceride lipase | 0,3067 | 2,027 | 0 | 0 | ≤0.001 |
| SLCO1B3 | Solute carrier organic anion transporter family member 1B3 | 0,3069 | 2,027 | 1,63E-11 | 8,08E-10 | NS |
| ANOS1 | Anosmin 1 | 0,3058 | 2,022 | 0 | 0 | ≤0.001 |
| FCGR2A | Fc fragment of igg, low affinity iia, receptor (CD32) | 0,3053 | 2,02 | 0 | 0 | ≤0.001 |
| CDK1 | Cyclin-dependent kinase 1 | 0,3047 | 2,017 | 0 | 0 | ≤0.001 |
| MMP9 | Matrix metallopeptidase 9 | 0,304 | 2,014 | 4,44E-16 | 2,53E-14 | ≤0.001 |
| ESM1 | Endothelial cell-specific molecule 1 | 0,3016 | 2,003 | 0 | 0 | ≤0.001 |
| CRIP1 | Cysteine-rich protein 1 (intestinal) | 0,3016 | 2,002 | 0 | 0 | ≤0.001 |
| ERO1A | Endoplasmic reticulum oxidoreductase alpha | 0,3012 | 2,001 | 0 | 0 | ≤0.001 |
| FHL2 | Four and a half LIM domains 2 | 0,3011 | 2 | 0 | 0 | ≤0.001 |

|  | *DOWN-REGULATED GENES*  *PDAC vs. Adjacent Tissues* | | | | | *GENE EXPRESSION VALIDATION DATASETS*  *PDAC vs. Healthy*  *TCGA PAAD combined GTEx Data* |
| --- | --- | --- | --- | --- | --- | --- |
| Gene Symbol | **Gene name** | **Logratio combined** | **Fold change combined** | ***p*-value** | ***FDR*** | ***p*-value** |
| ALB | albumin | -0,8658 | 7,342 | 0 | 0 | ≤0.001 |
| SERPINI2 | serpin peptidase inhibitor, clade I (pancpin), member 2 | -0,783 | 6,068 | 8,88E-16 | 4,96E-14 | ≤0.001 |
| PNLIPRP1 | pancreatic lipase-related protein 1 | -0,7683 | 5,866 | 9,40E-12 | 4,71E-10 | ≤0.001 |
| ERP27 | endoplasmic reticulum protein 27 | -0,7367 | 5,454 | 6,66E-16 | 3,75E-14 | ≤0.001 |
| PNLIPRP2 | pancreatic lipase-related protein 2 | -0,7359 | 5,444 | 3,66E-12 | 1,85E-10 | ≤0.001 |
| CTRL | chymotrypsin-like | -0,7199 | 5,247 | 3,55E-15 | 1,94E-13 | ≤0.001 |
| PDIA2 | protein disulfide isomerase family A member 2 | -0,7025 | 5,041 | 0 | 0 | ≤0.001 |
| GP2 | glycoprotein 2 (zymogen granule membrane) | -0,7005 | 5,018 | 3,06E-11 | 1,51E-09 | ≤0.001 |
| CELA2B | chymotrypsin like elastase family member 2B | -0,685 | 4,842 | 7,62E-12 | 3,83E-10 | ≤0.001 |
| IAPP | islet amyloid polypeptide | -0,6768 | 4,751 | 8,88E-16 | 4,96E-14 | ≤0.001 |
| CUZD1 | CUB and zona pellucida-like domains 1 | -0,6614 | 4,585 | 4,96E-10 | 2,41E-08 | ≤0.001 |
| CPA2 | carboxypeptidase A2 | -0,6564 | 4,533 | 3,17E-10 | 1,54E-08 | ≤0.001 |
| CTRC | chymotrypsin C (caldecrin) | -0,6538 | 4,507 | 4,22E-10 | 2,05E-08 | ≤0.001 |
| TMED6 | transmembrane p24 trafficking protein 6 | -0,6468 | 4,434 | 0 | 0 | ≤0.001 |
| CELA2A | chymotrypsin like elastase family member 2A | -0,6447 | 4,412 | 1,06E-09 | 5,13E-08 | ≤0.001 |
| EGF | epidermal growth factor | -0,644 | 4,405 | 0 | 0 | ≤0.001 |
| ANPEP | alanyl (membrane) aminopeptidase | -0,642 | 4,385 | 0 | 0 | ≤0.001 |
| AQP8 | aquaporin 8 | -0,6248 | 4,215 | 0 | 0 | ≤0.001 |
| KIAA1324 | KIAA1324 | -0,613 | 4,102 | 0 | 0 | ≤0.001 |
| CLPS | colipase | -0,6108 | 4,081 | 1,98E-09 | 9,48E-08 | ≤0.001 |
| KLK1 | kallikrein 1 | -0,6076 | 4,052 | 2,44E-15 | 1,33E-13 | ≤0.001 |
| CEL | carboxyl ester lipase | -0,5999 | 3,98 | 1,83E-09 | 8,78E-08 | ≤0.001 |
| FGL1 | fibrinogen like 1 | -0,5795 | 3,798 | 0 | 0 | ≤0.001 |
| RBPJL | recombination signal binding protein for immunoglobulin kappa J region-like | -0,5783 | 3,787 | 0 | 0 | ≤0.001 |
| SYCN | syncollin | -0,5711 | 3,725 | 5,44E-09 | 2,59E-07 | ≤0.001 |
| TRHDE | thyrotropin-releasing hormone degrading enzyme | -0,5682 | 3,7 | 0 | 0 | ≤0.001 |
| PLA2G1B | phospholipase A2 group IB | -0,5636 | 3,661 | 1,11E-08 | 5,28E-07 | ≤0.001 |
| PM20D1 | peptidase M20 domain containing 1 | -0,5591 | 3,623 | 0 | 0 | ≤0.001 |
| NR5A2 | nuclear receptor subfamily 5 group A member 2 | -0,5542 | 3,582 | 0 | 0 | ≤0.001 |
| GNMT | glycine N-methyltransferase | -0,5524 | 3,568 | 0 | 0 | ≤0.001 |
| ERO1B | endoplasmic reticulum oxidoreductase beta | -0,5491 | 3,541 | 0 | 0 | ≤0.001 |
| AOX1 | aldehyde oxidase 1 | -0,5387 | 3,457 | 0 | 0 | ≤0.001 |
| GATM | glycine amidinotransferase  (L-arginine:glycine amidinotransferase) | -0,5323 | 3,406 | 5,73E-14 | 3,03E-12 | ≤0.001 |
| PAIP2B | poly(A) binding protein interacting protein 2B | -0,5281 | 3,373 | 0 | 0 | ≤0.001 |
| CELP | carboxyl ester lipase pseudogene | -0,5252 | 3,351 | 1,11E-11 | 5,56E-10 | - |
| F11 | coagulation factor XI | -0,5127 | 3,256 | 0 | 0 | ≤0.001 |
| GUCA1C | guanylate cyclase activator 1C | -0,5091 | 3,23 | 0 | 0 | ≤0.001 |
| PDK4 | pyruvate dehydrogenase kinase, isozyme 4 | -0,5081 | 3,222 | 0 | 0 | ≤0.001 |
| NRG4 | neuregulin 4 | -0,5034 | 3,187 | 0 | 0 | ≤0.001 |
| PNLIP | pancreatic lipase | -0,5024 | 3,18 | 8,47E-07 | 3,92E-05 | ≤0.001 |
| CPA1 | carboxypeptidase A1 | -0,5021 | 3,178 | 4,99E-07 | 2,32E-05 | ≤0.001 |
| GSTA2 | glutathione S-transferase alpha 2 | -0,4928 | 3,11 | 2,22E-16 | 1,27E-14 | ≤0.001 |
| SPX | spexin hormone | -0,4924 | 3,107 | 0 | 0 | ≤0.001 |
| SLC16A10 | solute carrier family 16 (aromatic amino acid transporter), member 10 | -0,479 | 3,013 | 0 | 0 | ≤0.001 |
| CELA3A | chymotrypsin like elastase family member 3A | -0,4738 | 2,977 | 2,67E-07 | 1,24E-05 | ≤0.001 |
| GPHA2 | glycoprotein hormone alpha 2 | -0,4722 | 2,966 | 0 | 0 | ≤0.001 |
| SLC39A5 | solute carrier family 39 (zinc transporter), member 5 | -0,4709 | 2,957 | 0 | 0 | ≤0.001 |
| SEL1L | sel-1 suppressor of lin-12-like (C. elegans) | -0,4645 | 2,914 | 0 | 0 | ≤0.001 |
| MT1G | metallothionein 1G | -0,4625 | 2,9 | 0 | 0 | ≤0.001 |
| ACADL | acyl-CoA dehydrogenase, long chain | -0,4618 | 2,896 | 0 | 0 | ≤0.001 |
| BACE1 | beta-site APP-cleaving enzyme 1 | -0,4578 | 2,869 | 0 | 0 | ≤0.001 |
| SLC43A1 | solute carrier family 43 (amino acid system L transporter), member 1 | -0,4559 | 2,857 | 0 | 0 | ≤0.001 |
| PSAT1 | phosphoserine aminotransferase 1 | -0,4558 | 2,856 | 0 | 0 | ≤0.001 |
| PRSS3 | protease, serine 3 | -0,4462 | 2,794 | 7,26E-11 | 3,56E-09 | ≤0.001 |
| CELA3B | chymotrypsin like elastase family member 3B | -0,445 | 2,786 | 1,11E-05 | 0,000505 | ≤0.001 |
| C5 | complement component 5 | -0,441 | 2,761 | 0 | 0 | ≤0.001 |
| DPP10 | dipeptidyl-peptidase 10 (inactive) | -0,4399 | 2,754 | 0 | 0 | ≤0.001 |
| REG1CP | regenerating family member 1 gamma, pseudogene | -0,4329 | 2,709 | 9,37E-06 | 0,000426 | - |
| PAK3 | p21 protein (Cdc42/Rac)-activated kinase 3 | -0,4287 | 2,683 | 2,22E-16 | 1,27E-14 | ≤0.001 |
| LGALS2 | lectin, galactoside-binding, soluble, 2 | -0,4236 | 2,652 | 0 | 0 | NS |
| GSTA1 | glutathione S-transferase alpha 1 | -0,423 | 2,648 | 0 | 0 | ≤0.001 |
| NRCAM | neuronal cell adhesion molecule | -0,4213 | 2,638 | 0 | 0 | ≤0.001 |
| FAM3B | family with sequence similarity 3 member B | -0,4184 | 2,621 | 5,27E-13 | 2,72E-11 | NS |
| CBS | cystathionine-beta-synthase | -0,4182 | 2,62 | 0 | 0 | ≤0.001 |
| KCNJ16 | potassium channel, inwardly rectifying subfamily J, member 16 | -0,4174 | 2,615 | 5,40E-10 | 2,62E-08 | ≤0.001 |
| AZGP1 | alpha-2-glycoprotein 1, zinc-binding | -0,416 | 2,606 | 6,44E-14 | 3,40E-12 | ≤0.001 |
| HOMER2 | homer scaffolding protein 2 | -0,4133 | 2,59 | 0 | 0 | ≤0.001 |
| RNU6-287P | RNA, U6 small nuclear 287, pseudogene | -0,4115 | 2,58 | 1,91E-09 | 9,17E-08 | - |
| CTRB2 | chymotrypsinogen B2 | -0,4114 | 2,579 | 3,47E-06 | 0,00016 | ≤0.001 |
| TMEM97 | transmembrane protein 97 | -0,4093 | 2,566 | 1,73E-12 | 8,85E-11 | ≤0.001 |
| ABAT | 4-aminobutyrate aminotransferase | -0,4088 | 2,563 | 0 | 0 | ≤0.001 |
| NUCB2 | nucleobindin 2 | -0,4055 | 2,544 | 0 | 0 | NS |
| SLC16A12 | solute carrier family 16, member 12 | -0,4049 | 2,54 | 0 | 0 | ≤0.001 |
| LMO3 | LIM domain only 3 | -0,4028 | 2,528 | 0 | 0 | ≤0.001 |
| RN7SL186P | RNA, 7SL, cytoplasmic 186, pseudogene | -0,4019 | 2,523 | 6,28E-06 | 0,000287 | - |
| CTNND2 | catenin delta 2 | -0,4016 | 2,521 | 0 | 0 | ≤0.001 |
| SCGN | secretagogin, EF-hand calcium binding protein | -0,4009 | 2,517 | 5,04E-13 | 2,60E-11 | NS |
| EPB41L4B | erythrocyte membrane protein band 4.1 like 4B | -0,4001 | 2,512 | 0 | 0 | ≤0.001 |
| MT1M | metallothionein 1M | -0,4 | 2,512 | 0 | 0 | NS |
| OR8D4 | olfactory receptor, family 8, subfamily D, member 4 | -0,3999 | 2,511 | 1,79E-11 | 8,86E-10 | NS |
| PDZK1 | PDZ domain containing 1 | -0,3997 | 2,51 | 0 | 0 | NS |
| BNIP3 | BCL2/adenovirus E1B 19kDa interacting protein 3 | -0,3992 | 2,507 | 0 | 0 | ≤0.001 |
| RNU6-570P | RNA, U6 small nuclear 570, pseudogene | -0,3987 | 2,504 | 2,51E-09 | 1,20E-07 | - |
| EPHX2 | epoxide hydrolase 2, cytoplasmic | -0,3972 | 2,496 | 0 | 0 | ≤0.001 |
| IL22RA1 | interleukin 22 receptor, alpha 1 | -0,3962 | 2,49 | 6,66E-16 | 3,75E-14 | ≤0.001 |
| TPST2 | tyrosylprotein sulfotransferase 2 | -0,3951 | 2,483 | 0 | 0 | ≤0.001 |
| SLC7A2 | solute carrier family 7 (cationic amino acid transporter, y+ system), member 2 | -0,3942 | 2,478 | 0 | 0 | ≤0.001 |
| SLC4A4 | solute carrier family 4 (sodium bicarbonate cotransporter), member 4 | -0,3936 | 2,475 | 1,14E-07 | 5,30E-06 | ≤0.001 |
| COCH | cochlin | -0,3928 | 2,471 | 2,22E-16 | 1,27E-14 | ≤0.001 |
| SLC1A2 | solute carrier family 1 (glial high affinity glutamate transporter), member 2 | -0,3926 | 2,47 | 0 | 0 | ≤0.001 |
| MIR217 | microRNA 217 | -0,3909 | 2,46 | 1,28E-08 | 6,06E-07 | - |
| REG1B | regenerating family member 1 beta | -0,3888 | 2,448 | 0,000172 | 0,007779 | ≤0.001 |
| PRSS1 | protease, serine 1 | -0,3847 | 2,425 | 7,92E-06 | 0,000361 | ≤0.001 |
| BTG2 | BTG family member 2 | -0,3831 | 2,416 | 0 | 0 | ≤0.001 |
| DPEP1 | dipeptidase 1 (renal) | -0,383 | 2,416 | 0 | 0 | ≤0.001 |
| DNASE1 | deoxyribonuclease I | -0,3802 | 2,4 | 0 | 0 | ≤0.001 |
| PRSS3P2 | protease, serine 3 pseudogene 2 | -0,3802 | 2,4 | 6,11E-07 | 2,83E-05 | - |
| RGN | regucalcin | -0,3742 | 2,367 | 0 | 0 | ≤0.001 |
| CHRM3 | cholinergic receptor, muscarinic 3 | -0,3738 | 2,365 | 0 | 0 | ≤0.001 |
| PBLD | phenazine biosynthesis-like protein domain containing | -0,3704 | 2,346 | 0 | 0 | NS |
| TCEA3 | transcription elongation factor A (SII), 3 | -0,3696 | 2,342 | 0 | 0 | ≤0.001 |
| SLC17A4 | solute carrier family 17, member 4 | -0,3678 | 2,332 | 1,47E-14 | 7,85E-13 | ≤0.001 |
| P2RX1 | purinergic receptor P2X, ligand gated ion channel, 1 | -0,3662 | 2,324 | 0 | 0 | ≤0.001 |
| IMPA2 | inositol(myo)-1(or 4)-monophosphatase 2 | -0,3656 | 2,321 | 1,78E-15 | 9,77E-14 | ≤0.001 |
| FABP4 | fatty acid binding protein 4, adipocyte | -0,3627 | 2,305 | 9,34E-08 | 4,37E-06 | NS |
| FAM129A | family with sequence similarity 129 member A | -0,3607 | 2,295 | 0 | 0 | NS |
| TDH | L-threonine dehydrogenase (pseudogene) | -0,3604 | 2,293 | 1,78E-15 | 9,77E-14 | - |
| MT1F | metallothionein 1F | -0,3568 | 2,274 | 0 | 0 | NS |
| FAM46C | family with sequence similarity 46 member C | -0,3564 | 2,272 | 0 | 0 | ≤0.001 |
| TTN | titin | -0,3553 | 2,266 | 0 | 0 | NS |
| AQP12B | aquaporin 12B | -0,3525 | 2,252 | 7,07E-12 | 3,56E-10 | ≤0.001 |
| ALKAL2 | family with sequence similarity 150 member B | -0,3515 | 2,247 | 1,55E-15 | 8,59E-14 | ≤0.001 |
| AMY2A | amylase, alpha 2A (pancreatic) | -0,3513 | 2,245 | 4,62E-06 | 0,000212 | ≤0.001 |
| CPB1 | carboxypeptidase B1 (tissue) | -0,3497 | 2,237 | 5,59E-05 | 0,002525 | ≤0.001 |
| GRPR | gastrin releasing peptide receptor | -0,3496 | 2,237 | 2,22E-16 | 1,27E-14 | ≤0.001 |
| RN7SL386P | RNA, 7SL, cytoplasmic 386, pseudogene | -0,3488 | 2,232 | 1,27E-05 | 0,000574 | - |
| F8 | coagulation factor VIII, procoagulant component | -0,3487 | 2,232 | 0 | 0 | NS |
| MCOLN3 | mucolipin 3 | -0,3487 | 2,232 | 0 | 0 | ≤0.001 |
| ANKRD62 | ankyrin repeat domain 62 | -0,3485 | 2,231 | 2,78E-09 | 1,33E-07 | NS |
| BEX1 | brain expressed X-linked 1 | -0,3437 | 2,206 | 8,34E-12 | 4,19E-10 | NS |
| ARSE | arylsulfatase E (chondrodysplasia punctata 1) | -0,343 | 2,203 | 2,52E-13 | 1,31E-11 | NS |
| IGFBP2 | insulin like growth factor binding protein 2 | -0,3428 | 2,202 | 1,95E-14 | 1,04E-12 | NS |
| GRB14 | growth factor receptor bound protein 14 | -0,342 | 2,198 | 1,76E-12 | 8,97E-11 | ≤0.001 |
| GATA4 | GATA binding protein 4 | -0,3413 | 2,195 | 0 | 0 | ≤0.001 |
| PRLR | prolactin receptor | -0,3406 | 2,191 | 0 | 0 | NS |
| ADH1B | alcohol dehydrogenase 1B (class I), beta polypeptide | -0,3395 | 2,185 | 6,27E-10 | 3,03E-08 | NS |
| EPHX1 | epoxide hydrolase 1, microsomal (xenobiotic) | -0,3394 | 2,185 | 0 | 0 | NS |
| CCDC110 | coiled-coil domain containing 110 | -0,339 | 2,182 | 0 | 0 | ≤0.001 |
| GAMT | guanidinoacetate N-methyltransferase | -0,3387 | 2,181 | 0 | 0 | ≤0.001 |
| GPT2 | glutamic pyruvate transaminase (alanine aminotransferase) 2 | -0,3373 | 2,174 | 4,44E-16 | 2,53E-14 | ≤0.001 |
| SYBU | syntabulin (syntaxin-interacting) | -0,3368 | 2,171 | 0 | 0 | ≤0.001 |
| LIFR | leukemia inhibitory factor receptor alpha | -0,3344 | 2,16 | 0 | 0 | NS |
| REG1A | regenerating family member 1 alpha | -0,3328 | 2,152 | 4,49E-06 | 0,000206 | ≤0.001 |
| SEC11C | SEC11 homolog C, signal peptidase complex subunit | -0,3301 | 2,138 | 0 | 0 | NS |
| FKBP11 | FK506 binding protein 11 | -0,3296 | 2,136 | 0 | 0 | ≤0.001 |
| SLC30A2 | solute carrier family 30 (zinc transporter), member 2 | -0,3292 | 2,134 | 0 | 0 | ≤0.001 |
| GLS2 | glutaminase 2 | -0,3288 | 2,132 | 0 | 0 | ≤0.001 |
| KLB | klotho beta | -0,3284 | 2,13 | 0 | 0 | NS |
| C2CD4B | C2 calcium-dependent domain containing 4B | -0,3259 | 2,118 | 2,22E-16 | 1,27E-14 | ≤0.001 |
| CYB5A | cytochrome b5 type A (microsomal) | -0,3256 | 2,117 | 0 | 0 | NS |
| SLC39A8 | solute carrier family 39 (zinc transporter), member 8 | -0,3227 | 2,102 | 0 | 0 | NS |
| CCDC69 | coiled-coil domain containing 69 | -0,3221 | 2,1 | 0 | 0 | NS |
| CXCL12 | chemokine (C-X-C motif) ligand 12 | -0,3188 | 2,084 | 0 | 0 | NS |
| ACAT1 | acetyl-CoA acetyltransferase 1 | -0,3186 | 2,083 | 0 | 0 | NS |
| CFTR | cystic fibrosis transmembrane conductance regulator | -0,318 | 2,079 | 0,000198 | 0,008931 | NS |
| UGT2A3 | UDP glucuronosyltransferase 2 family, polypeptide A3 | -0,3167 | 2,073 | 1,43E-08 | 6,75E-07 | NS |
| GIPC2 | GIPC PDZ domain containing family member 2 | -0,3129 | 2,056 | 0 | 0 | NS |
| DCDC2 | doublecortin domain containing 2 | -0,3118 | 2,05 | 7,88E-08 | 3,69E-06 | NS |
| GAS2 | growth arrest specific 2 | -0,3115 | 2,049 | 0 | 0 | NS |
| CTH | cystathionine gamma-lyase | -0,3113 | 2,048 | 0 | 0 | NS |
| KIF1A | kinesin family member 1A | -0,3104 | 2,044 | 0 | 0 | ≤0.001 |
| RNF186 | ring finger protein 186 | -0,3098 | 2,041 | 0 | 0 | NS |
| RAB26 | RAB26, member RAS oncogene family | -0,3089 | 2,037 | 0 | 0 | ≤0.001 |
| C6 | complement component 6 | -0,3088 | 2,036 | 2,36E-08 | 1,11E-06 | NS |
| ACSM3 | acyl-CoA synthetase medium-chain family member 3 | -0,308 | 2,032 | 2,46E-11 | 1,21E-09 | NS |
| PROX1 | prospero homeobox 1 | -0,3077 | 2,031 | 8,88E-16 | 4,96E-14 | NS |
| SLC3A1 | solute carrier family 3 (amino acid transporter heavy chain), member 1 | -0,3077 | 2,031 | 3,41E-06 | 0,000157 | NS |
| BRSK2 | BR serine/threonine kinase 2 | -0,3064 | 2,025 | 0 | 0 | ≤0.001 |
| SIDT2 | SID1 transmembrane family member 2 | -0,3053 | 2,02 | 0 | 0 | NS |
| MT1H | metallothionein 1H | -0,3052 | 2,019 | 0 | 0 | ≤0.001 |
| LOC285097 | uncharacterized FLJ38379 | -0,3045 | 2,016 | 1,55E-15 | 8,59E-14 | - |
| TMEM52 | transmembrane protein 52 | -0,3044 | 2,015 | 1,42E-14 | 7,65E-13 | ≤0.001 |
| RNU6-776P | RNA, U6 small nuclear 776, pseudogene | -0,3042 | 2,015 | 2,13E-06 | 9,82E-05 | - |
| PDCD4 | programmed cell death 4 (neoplastic transformation inhibitor) | -0,3031 | 2,01 | 0 | 0 | ≤0.001 |
| ECI2 | enoyl-CoA delta isomerase 2 | -0,303 | 2,009 | 0 | 0 | NS |
| MAT1A | methionine adenosyltransferase I, alpha | -0,303 | 2,009 | 0 | 0 | ≤0.001 |
| PABPC4 | poly(A) binding protein, cytoplasmic 4 (inducible form) | -0,3023 | 2,006 | 0 | 0 | ≤0.001 |
| DTNA | dystrobrevin alpha | -0,3016 | 2,003 | 0 | 0 | ≤0.001 |
