# Supplementary material for: Integrated transcriptome meta-analysis of pancreatic ductal adenocarcinoma and matched adjacent pancreatic tissues
Source: PeerJ. 2020 Oct 27;8:e10141. doi: 10.7717/peerj.10141 (PMC7597628; doi:10.7717/peerj.10141)
Supplement: Supplemental Information 3 — Light colored rows: activated TFs, dark colored row: inhibited TF. [file peerj-08-10141-s003.docx]

| Transcription Factor | P.Value | E.Value | Q.Value | FDR Control (B-H) | Intersection | Target Genes |
| --- | --- | --- | --- | --- | --- | --- |
| TCF7 | 0.000e+0 | 0.000e+0 | 0.000e+0 | 8.475e-4 | 6 | 17 |
| CTNNB1 | 0.000e+0 | 0.000e+0 | 0.000e+0 | 1.695e-3 | 27 | 300 |
| SMAD3 | 0.000e+0 | 0.000e+0 | 0.000e+0 | 2.542e-3 | 14 | 55 |
| JUN | 1.600e-4 | 9.440e-3 | 2.360e-3 | 3.390e-3 | 10 | 88 |
| SMAD7 | 0.000e+0 | 0.000e+0 | 0.000e+0 | 8.475e-4 | 6 | 10 |

**Supp. Table 2. Table shows the predicted transcription factors activated or inhibited in PDAC based on the lists of up-regulated and down-regulated genes resulted from the meta-analysis.** *Light colored rows: activated TFs, dark colored row: inhibited TF.*
