# Supplementary material for: Integrated transcriptome meta-analysis of pancreatic ductal adenocarcinoma and matched adjacent pancreatic tissues
Source: PeerJ. 2020 Oct 27;8:e10141. doi: 10.7717/peerj.10141 (PMC7597628; doi:10.7717/peerj.10141)
Supplement: Supplemental Information 4 — NS: nonsignificant, p≥ 0.05; N/A: not available, p: p value, HR: Hazard ratio. Underlined HR values indicate an association with good outcome. [file peerj-08-10141-s004.doc]

**Supp. Table 3: The results of the Kaplan-Meier survival analysis of the identified DEGs in five external datasets.**

***NS: nonsignificant (p>0.05); N/A: not available, p: p value, HR: Hazard ratio. Underlined HR values indicate an association with good outcome.***

|  | **TCGA Pan-Cancer PDAC** |  |  | **GSE21501** |  |  |  | **GSE50827** |  |  |  | **GSE57495** |  |  |  | **GSE71729** |  |  |  |
| --- | --- | --- | --- | --- | --- | --- | --- | --- | --- | --- | --- | --- | --- | --- | --- | --- | --- | --- | --- |
| **Gene**  **Symbol** | **HR** | ***P*** | **FDR** | **HR** | **LCI (95%)** | **UCI**  **(95%)** | ***P*** | **HR** | **LCI (95%)** | **UCI (95%)** | ***P*** | **HR** | **LCI (95%)** | **UCI (95%)** | **P** | **HR** | **LCI (95%)** | **UCI (95%)** | ***P*** |
| **LAMC2** | **3,06** | **0,00011000** | **0,02** | **1,53** | **1,15** | **2,04** | **0,003** | **NS** |  |  |  | **1,33** | **1,03** | **1,74** | **0,03** | **1,24** | **1,05** | **1,46** | **0,01** |
| **KRT19** | **3,23** | **0,00007900** | **0,01** | **NS** |  |  |  | **NS** |  |  |  | **NS** |  |  |  | **1,22** | **1,01** | **1,48** | **0,04** |
| **ITGB6** | **2,59** | **0,00000490** | **0,01** | **1,39** | **1,15** | **1,67** | **0,001** | **NS** |  |  |  | **1,54** | **1,16** | **2,04** | **0,002** | **1,30** | **1,06** | **1,59** | **0,01** |
| **ITGA2** | **2,80** | **0,00001200** | **0,01** | **NS** |  |  |  | **NS** |  |  |  | **NS** |  |  |  | **NS** |  |  |  |
| **SERPINB5** | **2,89** | **0,00000540** | **0,01** | **1,37** | **1,09** | **1,72** | **0,01** | **NS** |  |  |  | **1,23** | **1,02** | **1,47** | **0,03** | **NS** |  |  |  |
| **TMPRSS4** | **2,20** | **0,00030000** | **0,05** | **NS** |  |  |  | **0,73** | **0,55** | **0,97** | **0,03** | **NS** |  |  |  | **NS** |  |  |  |
| **LAMB3** | **2,18** | **0,00030000** | **0,05** | **NS** |  |  |  | **NS** |  |  |  | **NS** |  |  |  | **1,23** | **1,04** | **1,46** | **0,01** |
| **S100P** | **2,75** | **0,00020000** | **0,05** | **NS** |  |  |  | **NS** |  |  |  | **NS** |  |  |  | **NS** |  |  |  |
| **CXCL5** | **2,49** | **0,00020000** | **0,05** | **NS** |  |  |  | **NS** |  |  |  | **NS** |  |  |  | **NS** |  |  |  |
| **KRT7** | **3,12** | **0,00007200** | **0,01** | **1,29** | **1,03** | **1,61** | **0,02** | **NS** |  |  |  | **1,46** | **1,12** | **1,89** | **0,004** | **NS** |  |  |  |
| **AHNAK2** | **4,18** | **0,00000440** | **0,01** | **NS** |  |  |  | **NS** |  |  |  | **NS** |  |  |  | **NS** |  |  |  |
| **ANO1** | **2,72** | **0,00020000** | **0,05** | **NS** |  |  |  | **NS** |  |  |  | **NS** |  |  |  | **NS** |  |  |  |
| **GPRC5A** | **2,64** | **0,00000460** | **0,01** | **NS** |  |  |  | **NS** |  |  |  | **1,26** | **1,00** | **1,59** | **0,05** | **NS** |  |  |  |
| **ANLN** | **2,38** | **0,00003600** | **0,01** | **NS** |  |  |  | **NS** |  |  |  | **NS** |  |  |  | **NS** |  |  |  |
| **SEMA3C** | **2,35** | **0,00004100** | **0,01** | **NS** |  |  |  | **NS** |  |  |  | **1,43** | **1,05** | **1,95** | **0,02** | **0,74** | **0,59** | **0,94** | **0,01** |
| **TGM2** | **2,14** | **0,00030000** | **0,05** | **NS** |  |  |  | **1,42** | **1,01** | **2,00** | **0,04** | **1,48** | **1,10** | **2,00** | **0,01** | **NS** |  |  |  |
| **LAMA3** | **3,86** | **0,00000360** | **0,01** | **1,50** | **1,16** | **1,95** | **0,002** | **NS** |  |  |  | **NS** |  |  |  | **1,57** | **1,21** | **2,04** | **0,001** |
| **DKK1** | **3,21** | **0,00010000** | **0,05** | **NS** |  |  |  | **NS** |  |  |  | **NS** |  |  |  | **NS** |  |  |  |
| **ECT2** | **2,27** | **0,00010000** | **0,02** | **NS** |  |  |  | **NS** |  |  |  | **NS** |  |  |  | **NS** |  |  |  |
| **SLC2A1** | **3,73** | **0,00004400** | **0,01** | **NS** |  |  |  | **NS** |  |  |  | **1,33** | **1,07** | **1,64** | **0,01** | **NS** |  |  |  |
| **TOP2A** | **2,49** | **0,00004900** | **0,01** | **NS** |  |  |  | **NS** |  |  |  | **NS** |  |  |  | **NS** |  |  |  |
| **COL17A1** | **2,19** | **0,00020000** | **0,02** | **NS** |  |  |  | **NS** |  |  |  | **1,20** | **1,02** | **1,41** | **0,03** | **NS** |  |  |  |
| **DHRS9** | **2,14** | **0,00030000** | **0,05** | **NS** |  |  |  | **NS** |  |  |  | **1,31** | **1,09** | **1,57** | **0,004** | **NS** |  |  |  |
| **ITGA3** | **2,58** | **0,00007500** | **0,02** | **NS** |  |  |  | **NS** |  |  |  | **NS** |  |  |  | **NS** |  |  |  |
| **MET** | **2,79** | **0,00000012** | **0,01** | **NS** |  |  |  | **NS** |  |  |  | **1,68** | **1,22** | **2,32** | **0,001** | **NS** |  |  |  |
| **ARNTL2** | **2,51** | **0,00000740** | **0,01** | **1,47** | **1,12** | **1,95** | **0,01** | **NS** |  |  |  | **NS** |  |  |  | **NS** |  |  |  |
| **IGF2BP3** | **3,55** | **0,00001000** | **0,01** | **1,17** | **1,01** | **1,35** | **0,04** | **NS** |  |  |  | **1,28** | **1,07** | **1,53** | **0,01** | **NS** |  |  |  |
| **AREG** | **2,82** | **0,00030000** | **0,05** | **NS** |  |  |  | **NS** |  |  |  | **N/A** |  |  |  | **NS** |  |  |  |
| **EFNB2** | **2,35** | **0,00008700** | **0,02** | **NS** |  |  |  | **NS** |  |  |  | **NS** |  |  |  | **NS** |  |  |  |
| **INPP4B** | **3,07** | **0,00010000** | **0,02** | **1,37** | **1,03** | **1,84** | **0,03** | **NS** |  |  |  | **1,24** | **1,02** | **1,51** | **0,03** | **NS** |  |  |  |
| **LIPH** | **2,38** | **0,00050000** | **0,05** | **NS** |  |  |  | **NS** |  |  |  | **NS** |  |  |  | **NS** |  |  |  |
| **ADAM9** | **2,39** | **0,00002800** | **0,01** | **NS** |  |  |  | **NS** |  |  |  | **NS** |  |  |  | **NS** |  |  |  |
| **IL1RN** | **2,39** | **0,00070000** | **0,05** | **1,29** | **1,04** | **1,60** | **0,02** | **NS** |  |  |  | **1,26** | **1,00** | **1,59** | **0,05** | **NS** |  |  |  |
| **EPSTI1** | **2,22** | **0,00020000** | **0,05** | **NS** |  |  |  | **NS** |  |  |  | **NS** |  |  |  | **1,45** | **1,04** | **2,04** | **0,03** |
| **ANXA2** | **2,50** | **0,00000840** | **0,01** | **1,61** | **1,03** | **2,53** | **0,04** | **NS** |  |  |  | **NS** |  |  |  | **NS** |  |  |  |
| **CENPF** | **2,18** | **0,00010000** | **0,05** | **NS** |  |  |  | **NS** |  |  |  | **NS** |  |  |  | **NS** |  |  |  |
| **ANTXR2** | **2,21** | **0,00010000** | **0,02** | **NS** |  |  |  | **NS** |  |  |  | **NS** |  |  |  | **NS** |  |  |  |
| **DDX60** | **2,14** | **0,00030000** | **0,05** | **NS** |  |  |  | **NS** |  |  |  | **NS** |  |  |  | **NS** |  |  |  |
| **DCBLD2** | **2,19** | **0,00020000** | **0,02** | **NS** |  |  |  | **NS** |  |  |  | **1,57** | **1,16** | **2,12** | **0,00** | **1,29** | **1,01** | **1,64** | **0,04** |
| **ANKRD22** | **2,04** | **0,00060000** | **0,05** | **NS** |  |  |  | **NS** |  |  |  | **NS** |  |  |  | **NS** |  |  |  |
| **AK4 (AK3L1)** | **2,26** | **0,00007400** | **0,02** | **NS** |  |  |  | **N/A** |  |  |  | **1,30** | **1,02** | **1,66** | **0,04** | **N/A** |  |  |  |
| **SAMD9** | **2,18** | **0,00020000** | **0,02** | **NS** |  |  |  | **NS** |  |  |  | **NS** |  |  |  | **NS** |  |  |  |
| **FGD6** | **3,30** | **0,00005600** | **0,02** | **NS** |  |  |  | **NS** |  |  |  | **NS** |  |  |  | **NS** |  |  |  |
| **OAS1** | **2,56** | **0,00030000** | **0,05** | **NS** |  |  |  | **NS** |  |  |  | **NS** |  |  |  | **NS** |  |  |  |
| **DLGAP5** | **2,56** | **0,00000990** | **0,01** | **NS** |  |  |  | **NS** |  |  |  | **NS** |  |  |  | **NS** |  |  |  |
| **FRMD6** | **2,33** | **0,00030000** | **0,05** | **NS** |  |  |  | **NS** |  |  |  | **1,65** | **1,15** | **2,37** | **0,01** | **NS** |  |  |  |
| **EPYC** | **2,24** | **0,00030000** | **0,05** | **NS** |  |  |  | **NS** |  |  |  | **NS** |  |  |  | **NS** |  |  |  |
| **S100A16** | **2,16** | **0,00020000** | **0,05** | **1,40** | **1,02** | **1,94** | **0,04** | **NS** |  |  |  | **NS** |  |  |  | **NS** |  |  |  |
| **ARHGAP42** | **2,06** | **0,00060000** | **0,05** | **NS** |  |  |  | **NS** |  |  |  | **NS** |  |  |  | **NS** |  |  |  |
| **MPZL2** | **2,30** | **0,00010000** | **0,02** | **NS** |  |  |  | **1,47** | **1,01** | **2,14** | **0,05** | **1,36** | **1,00** | **1,84** | **0,05** | **NS** |  |  |  |
| **PKM** | **2,52** | **0,00001000** | **0,01** | **1,88** | **1,19** | **2,97** | **0,01** | **N/A** |  |  |  | **N/A** |  |  |  | **N/A** |  |  |  |
| **IL1RAP** | **2,51** | **0,00010000** | **0,05** | **NS** |  |  |  | **NS** |  |  |  | **NS** |  |  |  | **1,56** | **1,03** | **2,37** | **0,03** |
| **TPX2** | **2,37** | **0,00003100** | **0,01** | **NS** |  |  |  | **NS** |  |  |  | **NS** |  |  |  | **NS** |  |  |  |
| **MKI67** | **2,28** | **0,00006700** | **0,02** | **NS** |  |  |  | **NS** |  |  |  | **NS** |  |  |  | **NS** |  |  |  |
| **SFTA2** | **2,39** | **0,00020000** | **0,02** | **NS** |  |  |  | **NS** |  |  |  | **1,37** | **1,13** | **1,64** | **0,00** | **NS** |  |  |  |
| **KCNN4** | **2,08** | **0,00050000** | **0,05** | **NS** |  |  |  | **NS** |  |  |  | **NS** |  |  |  | **NS** |  |  |  |
| **TRIM59** | **2,48** | **0,00001800** | **0,01** | **NS** |  |  |  | **NS** |  |  |  | **NS** |  |  |  | **NS** |  |  |  |
| **FAM83D** | **2,31** | **0,00004700** | **0,01** | **NS** |  |  |  | **NS** |  |  |  | **NS** |  |  |  | **NS** |  |  |  |
| **GPR87** | **3,37** | **0,00000460** | **0,01** | **NS** |  |  |  | **NS** |  |  |  | **1,15** | **1,00** | **1,33** | **0,04** | **NS** |  |  |  |
| **MGLL** | **2,17** | **0,00060000** | **0,05** | **NS** |  |  |  | **0,43** | **0,22** | **0,81** | **0,01** | **NS** |  |  |  | **NS** |  |  |  |
| **CDK1** | **2,65** | **0,00000410** | **0,01** | **N/A** |  |  |  | **N/A** |  |  |  | **NS** |  |  |  | **N/A** |  |  |  |
| **DTNA** | **0,42** | **0,00011000** | **0,01** | **NS** |  |  |  | **NS** |  |  |  | **NS** |  |  |  | **NS** |  |  |  |
