# Supplementary material for: Integrated transcriptome meta-analysis of pancreatic ductal adenocarcinoma and matched adjacent pancreatic tissues
Source: PeerJ. 2020 Oct 27;8:e10141. doi: 10.7717/peerj.10141 (PMC7597628; doi:10.7717/peerj.10141)
Supplement: Supplemental Information 5 [file peerj-08-10141-s005.docx]

**Supp. Table 4. Table shows the list of enriched categories and corresponding enriched genes for MCODE clusters in the protein-protein interaction network.**

|  | **Category** | **Term Name** | **Description** | **FDR P value** | **Enriched Genes** |
| --- | --- | --- | --- | --- | --- |
| **CLUSTER 1** | InterPro | IPR008160 | Collagen triple helix repeat | 2,08E-21 | [COL1A1, COL8A1, COL6A3, COL1A2, COL3A1, COL12A1, COL10A1, COL17A1, COL4A2, COL11A1, COL5A1, COL5A2, COL4A1] |
|  | GO Component | GO.0005788 | endoplasmic reticulum lumen | 3,03E-54 | [LGALS1, TIMP1, COL1A1, IGFBP5, COL8A1, CP, VCAN, TNC, COL6A3, ALB, COL1A2, COL3A1, APOL1, COL12A1, FBN1, COL10A1, COL17A1, FN1, COL4A2, F5, COL11A1, COL5A1, COL5A2, COL4A1, IGFBP3, MSLN, SPP1, MATN3, LTBP1, SERPINA1, SERPINH1] |
|  | GO Function | GO.0005201 | extracellular matrix structural constituent | 5,45E-11 | [VCAN, COL1A2, COL3A1, FBN1, COL4A2, COL11A1, COL4A1, MATN3] |
|  | GO Process | GO.0043062 | extracellular structure organization | 8,03E-27 | [TIMP1, COL1A1, COL8A1, VCAN, TNC, COL6A3, ALB, COL1A2, COL3A1, COL12A1, FBN1, COL10A1, FN1, COL4A2, COL11A1, COL5A1, COL5A2, COL4A1, SPP1, MATN3, SERPINH1] |
|  | KEGG Pathways | hsa04974 | Protein digestion and absorption | 6,59E-19 | [COL1A1, COL6A3, COL1A2, COL3A1, COL12A1, COL10A1, COL17A1, COL4A2, COL11A1, COL5A1, COL5A2, COL4A1] |
|  | KEGG Pathways | hsa04512 | ECM-receptor interaction | 8,69E-10 | [COL1A1, TNC, COL6A3, COL1A2, COL4A2, COL4A1, SPP1] |
|  | KEGG Pathways | hsa04510 | Focal adhesion | 2,12E-07 | [COL1A1, TNC, COL6A3, COL1A2, COL4A2, COL4A1, SPP1] |
|  | SMART | SM00038 | Fibrillar collagens C-terminal domain | 3,21E-12 | [COL1A1, COL1A2, COL3A1, COL11A1, COL5A1, COL5A2] |
|  | UniProt Keywords | KW-0272 | Extracellular matrix | 4,48E-26 | [LGALS1, COL1A1, COL8A1, VCAN, TNC, COL6A3, COL1A2, COL3A1, COL12A1, FBN1, COL10A1, COL17A1, COL4A2, COL11A1, COL5A1, COL5A2, COL4A1, LTBP1, SERPINA1] |
| **CLUSTER 2** | GO Function | GO.0008009 | chemokine activity | 3,02E-11 | [C5, CXCL5, CXCL10, CXCL8, CCL20, CXCL12] |
|  | GO Process | GO.0060326 | cell chemotaxis | 3,43E-11 | [C5, CXCL5, CXCL10, CXCL8, CCL20, ANXA1, CXCL12, CXCR4] |
|  | KEGG Pathways | hsa04062 | Chemokine signaling pathway | 2,82E-10 | [GNB4, CXCL5, CXCL10, CXCL8, CCL20, CXCL12, CXCR4] |
|  | SMART | SM00199 | Intercrine alpha family (small cytokine C-X-C) (chemokine CXC). | 5,01E-10 | [CXCL5, CXCL10, CXCL8, CCL20, CXCL12] |
|  | InterPro | IPR001811 | Chemokine interleukin-8-like domain | 2,63E-09 | [CXCL5, CXCL10, CXCL8, CCL20, CXCL12] |
|  | GO Component | GO.0005576 | extracellular region | 7,08E-06 | [C5, NMU, CXCL5, INSL5, CXCL10, CXCL8, CCL20, ANXA1, GABBR1, CXCL12] |
|  | UniProt Keywords | KW-0964 | Secreted | 1,71E-07 | [C5, NMU, CXCL5, INSL5, CXCL10, CXCL8, CCL20, ANXA1, GABBR1, CXCL12] |
|  | UniProt Keywords | KW-0395 | Inflammatory response | 8,42E-07 | [C5, CXCL10, CXCL8, CCL20, ANXA1] |
| **CLUSTER 3** | UniProt Keywords | KW-0131 | Cell cycle | 6,28E-10 | [DLGAP5, CCNB1, MELK, TPX2, CENPF, ASPM, MKI67, CDK1] |
|  | GO Process | GO.0007049 | cell cycle | 1,53E-08 | [DLGAP5, CCNB1, MELK, TPX2, CENPF, ASPM, MKI67, CDK1, TOP2A] |
|  | GO Component | GO.0005819 | spindle | 2,03E-07 | [DLGAP5, CCNB1, TPX2, CENPF, ASPM, CDK1] |
|  | GO Function | GO.0035173 | histone kinase activity | 0,0029 | [CCNB1, CDK1] |
|  | KEGG Pathways | hsa04110 | Cell cycle | 0,0049 | [CCNB1, CDK1] |
| **CLUSTER 4** | GO Function | GO.0001730 | 2'-5'-oligoadenylate synthetase activity | 8,91E-05 | [OAS2, OAS1] |
|  | GO Process | GO.0060337 | type I interferon signaling pathway | 1,79E-11 | [BST2, OAS2, XAF1, RSAD2, OAS1, IFI27] |
|  | InterPro | IPR024644 | Interferon-induced protein 44 family | 1,58E-05 | [IFI44, IFI44L] |
|  | InterPro | IPR024644 | Interferon-induced protein 44 family | 1,58E-05 | [IFI44, IFI44L] |
|  | KEGG Pathways | hsa04621 | NOD-like receptor signaling pathway | 0,0031 | [OAS2, OAS1] |
|  | UniProt Keywords | KW-0051 | Antiviral defense | 1,20E-10 | [BST2, OAS2, IFI44L, RSAD2, OAS1, IFI27] |
|  | UniProt Keywords | KW-0399 | Innate immunity | 1,50E-06 | [BST2, OAS2, RSAD2, OAS1, IFI27] |
|  | UniProt Keywords | KW-0548 | Nucleotidyltransferase | 0,0055 | [OAS2, OAS1] |
| **CLUSTER 5** | GO Component | GO.0005882 | intermediate filament | 5,18E-11 | [KRT23, KRT6B, KRT17, KRT7, KRT19, KRT6A] |
|  | GO Function | GO.0005198 | structural molecule activity | 2,34E-08 | [KRT23, KRT6B, KRT17, KRT7, KRT19, KRT6A] |
|  | GO Process | GO.0070268 | Cornification | 7,31E-12 | [KRT23, KRT6B, KRT17, KRT7, KRT19, KRT6A] |
|  | InterPro | IPR001664 | Intermediate filament protein | 8,42E-15 | [KRT23, KRT6B, KRT17, KRT7, KRT19, KRT6A] |
|  | KEGG Pathways | hsa04915 | Estrogen signaling pathway | 6,46E-06 | [KRT23, KRT17, KRT19] |
|  | KEGG Pathways | hsa04915 | Estrogen signaling pathway | 6,46E-06 | [KRT23, KRT17, KRT19] |
|  | UniProt Keywords | KW-0403 | Intermediate filament | 7,07E-14 | [KRT23, KRT6B, KRT17, KRT7, KRT19, KRT6A] |
| **CLUSTER 6** | GO Component | GO.0005604 | basement membrane | 7,82E-08 | [LAMA4, SPARC, LAMC2, LAMA3, LAMB3] |
|  | GO Component | GO.0043256 | laminin complex | 6,53E-07 | [LAMC2, LAMA3, LAMB3] |
|  | GO Component | GO.0044420 | extracellular matrix component | 6,53E-07 | [LAMA4, LAMC2, LAMA3, LAMB3] |
|  | GO Function | GO.0005102 | signaling receptor binding | 0,0268 | [ITGA3, ITGB4, LAMA4, EGF, LAMA3] |
|  | GO Function | GO.0005518 | collagen binding | 0,0268 | [ITGA3, SPARC] |
|  | GO Function | GO.0046934 | phosphatidylinositol-4,5-bisphosphate 3-kinase activity | 0,0268 | [EGF, MET] |
|  | GO Process | GO.0030198 | extracellular matrix organization | 1,52E-08 | [ITGA3, ITGB4, LAMA4, SPARC, LAMC2, LAMA3, LAMB3] |
|  | GO Process | GO.0031581 | hemidesmosome assembly | 1,93E-08 | [ITGB4, LAMC2, LAMA3, LAMB3] |
|  | GO Process | GO.0030334 | regulation of cell migration | 1,86E-06 | [ITGA3, LAMA4, SPARC, LAMC2, EGF, MET, LAMA3] |
|  | InterPro | IPR002049 | Laminin EGF domain | 2,05E-07 | [LAMA4, LAMC2, LAMA3, LAMB3] |
|  | InterPro | IPR013032 | EGF-like, conserved site | 6,58E-05 | [ITGB4, EGF, LAMA3, LAMB3] |
|  | KEGG Pathways | hsa04510 | Focal adhesion | 2,79E-13 | [ITGA3, ITGB4, LAMA4, LAMC2, EGF, MET, LAMA3, LAMB3] |
|  | KEGG Pathways | hsa04151 | PI3K-Akt signaling pathway | 1,21E-11 | [ITGA3, ITGB4, LAMA4, LAMC2, EGF, MET, LAMA3, LAMB3] |
|  | KEGG Pathways | hsa04512 | ECM-receptor interaction | 2,23E-11 | [ITGA3, ITGB4, LAMA4, LAMC2, LAMA3, LAMB3] |

| **CLUSTER 7** | GO Component | GO.0005796 | Golgi lumen | 4,84E-07 | [MUC17, MUC16, MUC4, MUC13] |
| --- | --- | --- | --- | --- | --- |
|  | GO Function | GO.0030197 | extracellular matrix constituent, lubricant activity | 3,29E-05 | [MUC17, MUC4] |
|  | GO Process | GO.0016266 | O-glycan processing | 9,67E-14 | [GALNT5, MUC17, GCNT3, MUC16, MUC4, MUC13] |
|  | InterPro | IPR000082 | SEA domain | 4,77E-07 | [MUC17, MUC16, MUC13] |
|  | KEGG Pathways | hsa00512 | Mucin type O-glycan biosynthesis | 7,74E-05 | [GALNT5, GCNT3] |
|  | SMART | SM00200 | Domain found in sea urchin sperm protein, enterokinase, agrin | 7,61E-08 | [MUC17, MUC16, MUC13] |
|  | SMART | SM00001 | EGF domain, unclasssified subfamily | 4,39E-06 | [MUC17, MUC4, MUC13] |
|  | UniProt Keywords | KW-1015 | Disulfide bond | 4,60E-04 | [GALNT5, MUC17, GCNT3, MUC16, MUC4, MUC13] |
|  | UniProt Keywords | KW-0325 | Glycoprotein | 0,0013 | [GALNT5, MUC17, GCNT3, MUC16, MUC4, MUC13] |
| **CLUSTER 8** | GO Component | GO.0031225 | anchored component of membrane | 2,87E-09 | [CEACAM5, THY1, CD109, GP2, NTM] |
|  | GO Function | GO.0034235 | GPI anchor binding | 2,74E-05 | [CEACAM5, THY1] |
|  | InterPro | IPR013783 | Immunoglobulin-like fold | 1,70E-04 | [CEACAM5, THY1, CD109, NTM] |
|  | SMART | SM00409 | Immunoglobulin | 3,30E-04 | [CEACAM5, THY1, NTM] |
|  | SMART | SM00409 | Immunoglobulin | 3,30E-04 | [CEACAM5, THY1, NTM] |
| **CLUSTER 9** | GO Component | GO.0035579 | specific granule membrane | 2,57E-10 | [CEACAM1, P2RX1, OLR1, PLAUR, PLAU] |
|  | GO Process | GO.0043312 | neutrophil degranulation | 5,37E-06 | [CEACAM1, P2RX1, OLR1, PLAUR, PLAU] |
|  | GO Process | GO.0042060 | wound healing | 3,07E-05 | [CEACAM1, P2RX1, PLAUR, PLAU] |
|  | KEGG Pathways | hsa05205 | Proteoglycans in cancer | 0,0054 | [PLAUR, PLAU] |
|  | GO Process | GO.0042058 | regulation of epidermal growth factor receptor signaling pathway | 0,0021 | [CEACAM1, PLAUR] |
