# Supplementary material for: Integrated transcriptome meta-analysis of pancreatic ductal adenocarcinoma and matched adjacent pancreatic tissues
Source: PeerJ. 2020 Oct 27;8:e10141. doi: 10.7717/peerj.10141 (PMC7597628; doi:10.7717/peerj.10141)
Supplement: Supplemental Information 6 — Lines with white color represent the positively related pathways while lines with gray color represent the negatively related pathways. Size is the number of overlapping genes between categories and the differentially expressed gene list. P<0.05 and FDR<0.05 are accepted as statistically significant. P and/or FDR values <2.220446e-16 were shown as 0e+00. NS: Normalized enrichment score, ES: Enrichment Score, LEN: Leading Edge Number. [file peerj-08-10141-s006.docx]

**Supp. Table 5. Results of the Pathway Enrichment Analysis.** Table represents the statistics of the enriched pathways and the leading edge genes. Lines with white color represent the positively related pathways while lines with grey color represent the negatively related pathways.  Size is the number of overlapping genes between categories and the differentially expressed gene list. *P*˂0.05 and *FDR*˂0.05 are accepted as statistically significant. *P* and/or *FDR* values ˂2.220446e-16 were shown as 0e+00*. NS: Normalized enrichment score, ES: Enrichment Score, LEN:* Leading Edge Number.

| **Pathway Database** | **Gene Set** | **Description** | **Size** | **LEN** | **ES** | **NES** | **P Value** | **FDR** |
| --- | --- | --- | --- | --- | --- | --- | --- | --- |
| **KEGG** | hsa04512 | ECM-Receptor Interaction | 18 | 11 | 0.6 | 2.5 | 0e+00 | 0e+00 |
|  | hsa04151 | PI3K-Akt Signalling Pathway | 24 | 9 | 0.47 | 2.11 | 0e+00 | 4.8e-03 |
|  | hsa04510 | Focal Adhesion | 22 | 9 | 0.49 | 2.15 | 3.18e-03 | 5.85e-03 |
|  | hsa05200 | Pathways in Cancer | 24 | 15 | 0.39 | 1.79 | 2.08e-02 | 2.6e-02 |
|  | hsa04972 | Pancreatic Secretion | 19 | 18 | -0.77 | -3.76 | 0e+00 | 0e+00 |
|  | hsa01100 | Metabolic Pathways | 47 | 24 | 0.27 | -1.82 | 1.25e-02 | 1.51e-02 |
| **PANTHER** | P00034 | Integrin Signalling Pathway | 22 | 17 | 0.72 | 3.23 | 0e+00 | 0e+00 |

| **WIKIPATHWAY** | WP306 | Focal Adhesion | 26 | 12 | 0.55 | 2.6 | 0e+00 | 0e+00 |
| --- | --- | --- | --- | --- | --- | --- | --- | --- |
|  | [WP3932](http://www.webgestalt.org/2017/results/Project_wg_result1548319414/Report_wg_result1548319414.html#WP3932) | Focal Adhesion-PI3K-Akt-mTOR-signaling pathway | 28 | 12 | 0.52 | 2.56 | 0e+00 | 0e+00 |
|  | [WP4172](http://www.webgestalt.org/2017/results/Project_wg_result1548319414/Report_wg_result1548319414.html#WP4172) | PI3K-Akt Signaling Pathway | 24 | 9 | 0.47 | 2.14 | 3.2e-03 | 1.23e-03 |
| **REACTOME** | [R-Hsa-1442490](http://www.webgestalt.org/2017/results/Project_wg_result1548153829/Report_wg_result1548153829.html#R-HSA-1442490) | Collagen Degradation | 23 | 14 | 0.58 | 2.64 | 0e+00 | 0e+00 |
|  | [R-Hsa-1474228](http://www.webgestalt.org/2017/results/Project_wg_result1548153829/Report_wg_result1548153829.html#R-HSA-1474228) | Degradation of the Extracellular Matrix | 32 | 20 | 0.6 | 3.04 | 0e+00 | 0e+00 |
|  | [R-Hsa-1474244](http://www.webgestalt.org/2017/results/Project_wg_result1548153829/Report_wg_result1548153829.html#R-HSA-1474244) | Extracellular Matrix Organization | 53 | 29 | 0.53 | 3.18 | 0e+00 | 0e+00 |
|  | [R-Hsa-1474290](http://www.webgestalt.org/2017/results/Project_wg_result1548153829/Report_wg_result1548153829.html#R-HSA-1474290) | Collagen Formation | 24 | 15 | 0.59 | 2.77 | 0e+00 | 0e+00 |
|  | [R-Hsa-1650814](http://www.webgestalt.org/2017/results/Project_wg_result1548153829/Report_wg_result1548153829.html#R-HSA-1650814) | Collagen Biosynthesis and Modifying Enzymes | 15 | 10 | 0.66 | 2.53 | 0e+00 | 0e+00 |
|  | [R-Hsa-2022090](http://www.webgestalt.org/2017/results/Project_wg_result1548153829/Report_wg_result1548153829.html#R-HSA-2022090) | Assembly of Collagen Fibrils and Other Multimeric Structures | 22 | 15 | 0.64 | 2.79 | 0e+00 | 0e+00 |
|  | [R-Hsa-216083](http://www.webgestalt.org/2017/results/Project_wg_result1548153829/Report_wg_result1548153829.html#R-HSA-216083) | Integrin Cell Surface Interactions | 19 | 13 | 0.64 | 2.69 | 0e+00 | 0e+00 |
|  | [R-Hsa-3000171](http://www.webgestalt.org/2017/results/Project_wg_result1548153829/Report_wg_result1548153829.html#R-HSA-3000171) | Non-Integrin Membrane-ECM Interactions | 17 | 12 | 0.69 | 2.83 | 0e+00 | 0e+00 |
|  | [R-Hsa-3000178](http://www.webgestalt.org/2017/results/Project_wg_result1548153829/Report_wg_result1548153829.html#R-HSA-3000178) | ECM Proteoglycans | 21 | 15 | 0.64 | 2.79 | 0e+00 | 0e+00 |
|  | [R-Hsa-109582](http://www.webgestalt.org/2017/results/Project_wg_result1548153829/Report_wg_result1548153829.html#R-HSA-109582) | Hemostasis | 35 | 13 | 0.34 | 1.8 | 1.36e-02 | 3.11e-02 |
|  | [R-Hsa-1430728](http://www.webgestalt.org/2017/results/Project_wg_result1548153829/Report_wg_result1548153829.html#R-HSA-1430728) | Metabolism | 80 | 45 | -0.26 | -2.08 | 6.67e-03 | 2.29e-02 |
